# Engineered extracellular vesicles for ischemic heart diseases: modification methods, targeted delivery strategies, and multi-modal therapies - A systematic review

**DOI:** 10.3389/fcvm.2026.1868328

**Published:** 2026-06-18

**Authors:** Sha Su, Yu Teng, Shaojiao Liu, Lei Wang, Mingjing Zhao

**Affiliations:** Key Laboratory of Chinese Internal Medicine of Ministry of Education and Beijing, Dongzhimen Hospital Affiliated to Beijing University of Chinese Medicine, Beijing, China

**Keywords:** engineered extracellular vesicles (engineered EVs), extracellular vesicles (EVs), IHD, multi-modal therapy, targeting strategy

## Abstract

**Background/purpose:**

Due to the complex pathological process of ischemic heart diseases (IHD), a single treatment strategy had limited efficacy. Multi-targeted synergy, precise delivery, and long-lasting effects were new directions for treatment. Engineering extracellular vesicles (EVs) had become a research hotspot in the field of IHD treatment due to their ability carrying therapeutic signaling molecules, precise tissue targeting capabilities, and excellent biocompatibilities. This systematic review focused on the modification methods, targeting strategies, and combined effects of multi-pathway synergy of engineered EVs in IHD treatment.

**Methods:**

Systematic searches were conducted in 8 databases. According to strict inclusion and exclusion criteria, the literature was screened, and relevant information was extracted based on the research purpose. Two researchers independently screened the literature, extracted information, and evaluated the quality of literatures.

**Results:**

A total of 50 animal studies were included. The existing studies mainly achieved the engineering modification of EVs through internal loading/knockdown, surface modification, membrane fusion, combination with biotechnological materials, and pre-treatment; and by using targeting peptides or specific antibodies modification, membrane fusion, and *in situ* cardiac delivery, to enhance their targeting enrichment abilities for ischemic myocardium. In terms of therapeutic effects, engineered EVs could exert beneficial effects on cardiac function through multiple pathways, such as alleviating myocardial fibrosis, inhibiting inflammatory responses, promoting angiogenesis, reducing cardiomyocyte apoptosis, and improving mitochondrial metabolism. The multi-modal therapy of engineered EVs presented a pyramid structure: improving cardiac function served as the foundation, ameliorating classical cardioprotective pathways constituted the primary pillars, and optimizing metabolic modulation represented supplementary.

**Conclusion:**

There was an intrinsic association between the multi-association therapeutic effects of engineered EVs and the modification methods. Currently, the modification strategies of engineered EVs formed a composite system of “ internal cargo loading/knockdown of core signaling molecules + surface modification and membrane fusion to enhance targeting specificity + combination with bioengineering materials for local sustained release”, which met the multiple needs of multi-targeted synergy, precise delivery, and long-lasting effects. This systematic review provided key theoretical basis and practical guidance for constructing a multifunctional EVs delivery system for treating IHD and accelerating its clinical translation and application.

**Systematic Review Registration:** https://www.crd.york.ac.uk/, identifier PROSPERO CRD420261393475.

## Graphical Abstract

**Figure:**
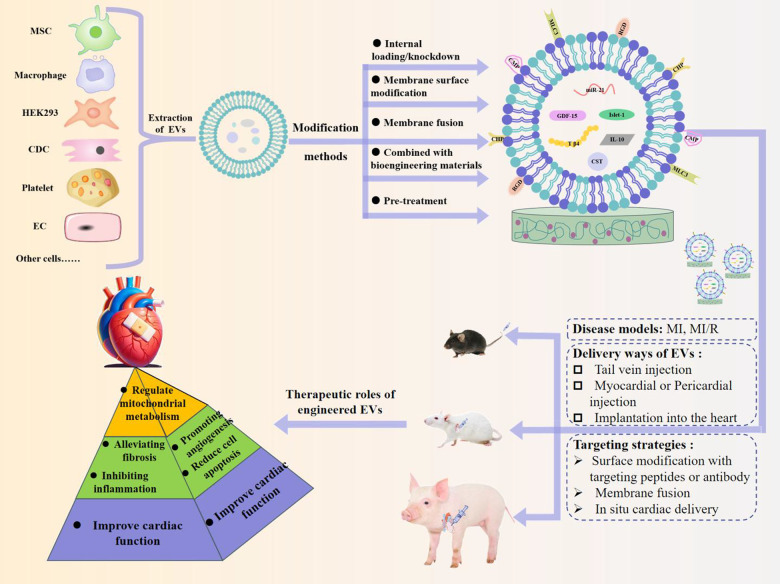
Graphical abstracts of this systematic review. CDC, cardiosphere-derived cell; EC, endothelial cell; EVs, extracellular vesicles; MI, myocardical infarection; MI/R, myocardical ischemia/reperfusion; MSC, mesenchymal stem cells.

## 1 Introduction

Ischemic heart disease (IHD) is a major cardiovascular disease. In 2021, approximately 254 million people worldwide suffered from IHD, resulting in nearly 9 million deaths. Since 1990, the number of patients had increased by 127%. The disease burden in countries with low socio-demographic indices is still on the rise, posing a significant threat to global public health (1, 2). Its incidence and mortality rates had consistently ranked first among all cardiovascular diseases (3). The pathological mechanisms of this disease are complex, and current medical treatments such as drug therapy, interventional therapy (such as stent implantation), and surgical procedures are difficult to achieve comprehensive and systematic myocardial repair. There are obvious limitations in clinical treatment (4–6). Extracellular vesicles (EVs) are natural biological carrier with diameters ranging from 30 to 1,000 nanometers. EVs are synthesized and secreted by various living cells and are nano-sized vesicles encapsulated by a lipid bilayer (7–10). EVs carry various bioactive substances such as proteins, nucleic acids (including mRNA, miRNA, lncRNA, etc.) and lipids. These substances travel through the bloodstream to various parts of the body and are delivered to target cells through membrane fusion and endocytosis, exerting important biological functions (11–16). Numerous studies had confirmed that EVs served as an important medium for intercellular information transmission and played a crucial regulatory role in the occurrence, development, and prognosis of IHD (17, 18). Engineered EVs refers to new vesicles obtained by artificial modification, optimization, or transformation based on natural EVs. They have unique advantages such as stronger targeting, more significant therapeutic effects, and longer-lasting effects. Currently, engineered EVs has gradually become a research hotspot in the field of new drug delivery carriers in biomedicine, and has emerged in the field of IHD treatment, with great clinical application potential and irreplaceable significance.

This systematic review conducted a comprehensive literature screening and detailed data extraction to systematically integrate the modification methods, targeting strategies, and therapeutic effects of engineered EVs, with a focus on analyzing the intrinsic relationship and matching rules between the multi-modal therapy and modification methods of engineered EVs. This review provided powerful scientific basis and practical guidance for subsequent optimization of engineered EVs design, improvement of therapeutic effects, and promotion of their clinical transformation, helping to innovate and develop technologies in the field of IHD treatment.

## 2 Methods

This systematic review report adhered to the PRISMA (Preferred Reporting Items for Systematic Reviews and Meta-Analyses) 2020 statement. PRISMA was an internationally recognized reporting guideline. The 2020 version (released in 2021) included 27 checklist items and a standardized literature screening flowchart, covering all aspects from the title, abstract, methods, results to discussion (19).

### 2.1 Search strategy

A literature search was conducted across 8 electronic databases, including PubMed, Web of Science, Embase, Cochrane, China National Knowledge Infrastructure, WanFang Database, VIP Database, and SinoMed. The search timeframe spanned from the inception of each database to March 2, 2026. The search strategy used the following general terms as MeSH terms or free terms: (“engineered membrane vesicle*” OR “engineered extracellular vesicle*” OR “engineered vesicle*” OR “engineered exosome*” OR “nanovesicle*” OR “nanoscale membrane vesicle*” OR “nanoscale extracellular vesicle*” OR “nanoscale vesicle*” OR “nanoscale exosome*”) AND (“myocardial ischemia” OR “angina pectoris” OR “myocardial infarction” OR “coronary atherosclerotic heart disease” OR “coronary disease*” OR “coronary heart disease” OR “CHD” OR “ischemic heart disease*” OR “angina” OR “MI”). No filters or restrictions were applied. The complete search strategy is detailed in the Supplementary Material. Additionally, review articles and references from identified studies were examined for further relevant studies.

### 2.2 Inclusion and exclusion criteria

Inclusion criteria: (1) studies involving modified or engineered EVs; (2) studies focusing on IHD; (3) studies conducted in animal or human models; (4) publications in peer-reviewed journals with no language restrictions.

Exclusion criteria: (1) articles with incomplete data; (2) duplicate publications; (3) reviews, meta-analyses, theses, and conference abstracts.

### 2.3 Quality assessment

The quality of the included studies was independently evaluated by two authors using SYRCLE's ROB tool for animal experiments. This tool assessed 10 items across 6 domains: sequence generation (selection bias), baseline characteristics (selection bias), allocation concealment (selection bias), random housing (performance bias), blinding (performance bias), random outcome assessment (detection bias), blinding (detection bias), incomplete outcomes data (attrition bias), selective outcome reporting (reporting bias), and other (other sources of bias). Each item was classified as “unclear” “low risk” or “high risk” (20). Disagreements were resolved by consensus after discussion with the corresponding author.

### 2.4 Data extraction

Two authors independently extracted data using standardized forms. The basic characteristics of the included studies were summarized, including the first author, publication year, country, parental cells of EVs, engineering modifications of EVs, animal sex and strain, disease types, methods of animal administration of engineered EVs, target cells of engineered EVs action, and therapeutic effects of engineered EVs. Disagreements were resolved through consensus with the corresponding author.

### 2.5 Data analysis

Firstly, the modification methods and signaling molecules of engineered EVs were summarized. Secondly, the *in vivo* targeting strategies of engineered EVs were discussed. Then, we summarized the therapeutic effects of engineered EVs in ischemic heart disease. However, due to the high heterogeneity of the included animal studies, a qualitative comprehensive analysis was applied in this systematic review. Compared with the model group, if the outcome index of the engineered EVs treatment group increased significantly, the result was recorded as “↑”, decreased markedly was recorded as “↓”. Finally, the relationship between the modification methods of engineered EVs and their therapeutic effects were jointly analyzed.

## 3 Results

### 3.1 Search results and study selection

**Figure 1:**
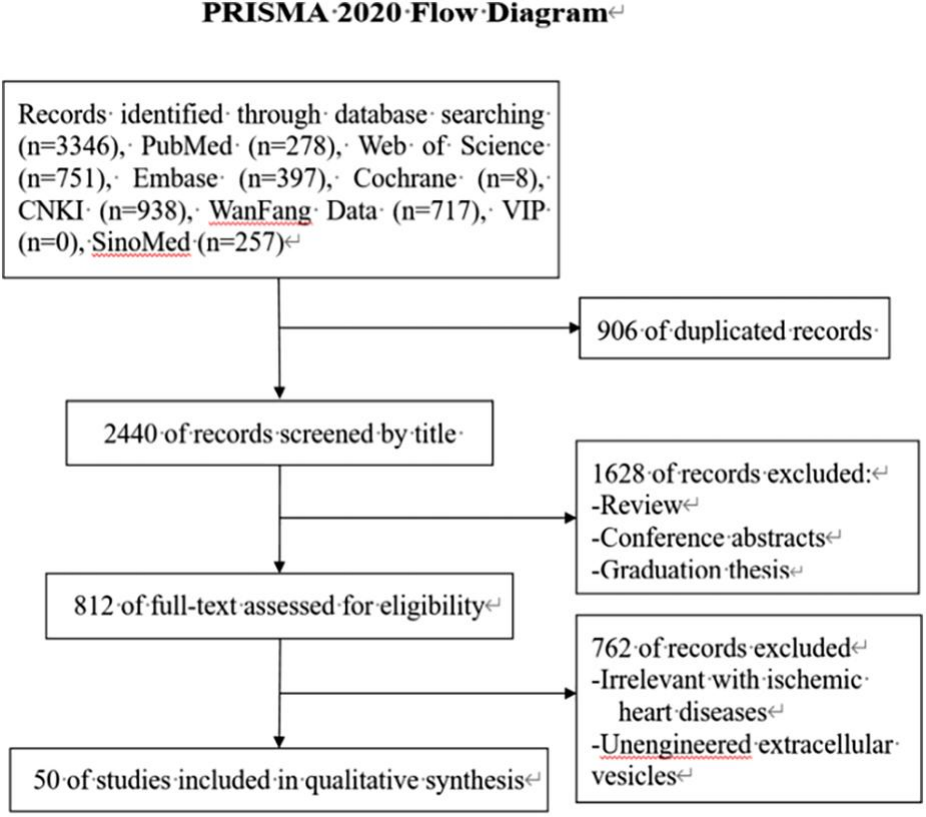
Flow diagram of the systematic literatures search.

A total of 3,346 records were identified from 8 electronic databases. After removing 906 duplicate entries, 2,440 potentially relevant studies remained. Titles and abstracts were subsequently screened, resulting in the exclusion of 1,628 studies. Of the remaining 812 studies, 762 were further excluded following a full-text review. Ultimately, 50 animal studies met the inclusion criteria and were included in this systematic review. The search process and study selection were detailed in the specific flowchart (Figure 1).

### 3.2 Characteristics of included studies

The systematic review incorporated 50 animal studies, with the summarized basic information presented in Table 1. The studies originated from China (38/50), the United States (5/50), Korea (2/50), Spain (2/50), India (2/50) and Singapore (1/50). The parental cells used for engineered EVs included mesenchymal stem cells (MSCs) (26/50), HEK 293 cells (4/50), adipose tissue-derived stem cells (ADSCs) (4/50), macrophages (3/50), cardiac-derived progenitor cells (CDCs) (3/50), neutrophils (3/50), other stem cells (3/50), endothelial cells (ECs) (1/50), fibroblasts (1/50), plant-derived vesicles (1/50), and H9c2 cells (1/50).

**Table 1:** Main data from the included studies.

**Author, year, country**	**Parental cells of EVs**	**Engineering modifications of EVs**	**Animal sex and strain/disease types**	**Methods of animal administration of engineering EVs**	**Target cells of engineering EVs action**	**Therapeutic effects of engineering EVs**
Mun, 2026, Korea (21)	MSCs	Loaded with IL-10 mRNA+ surface modificated with T peptide, anti-CD63 and MLC3 antibodies	Male (C57BL/6 mice)/MI	Tail vein injection	CMs, macrophages	Improve cardiac function, alleviate cardiac fibrosis and inflammation, inhibit cell apoptosis.
Luo, 2026, China (22)	HEK 293T cells	Loaded with Ndufs1 and VegfA mRNA + surface modificated with IMTP and Cx43	Male (C57BL/6 mice)/MI	Tail vein injection	CMs, ECs, macrophages	Improve cardiac function, alleviate cardiac fibrosis and inflammation, promote angiogenesis and mitochondrial metabolism.
Liu, 2026, China (23)	ADSCs, Macrophages	Cholesterol-modified macrophage membrane fusion	Male SD rat/MI	Tail vein injection	CMs, macrophages	Improve cardiac function, alleviate cardiac fibrosis and inflammation, promote angiogenesis, inhibit cell apoptosis.
Zhou, 2025, China (24)	Gouqi	Encapsulated within fibrin gels	Male (C57BL/6 mice)/MI	Myocardial injection	CMs	Improve cardiac function, alleviate cardiac fibrosis and cell apoptosis, promote angiogenesis and mitochondrial metabolism.
Zhao, 2025, China (25)	MSCs	Pre-treatment with ginsenoside Rg1	Male SD rat/(MI/R)	Myocardial injection	CMs	Improve cardiac function, alleviate cardiac fibrosis and cell apoptosis, promote mitochondrial metabolism.
Yang, 2025, China (26)	MSCs	Loaded with SIRT3 and Insulin	Male SD rat/(MI/R)	Myocardial injection	CMs	Improve cardiac function, alleviate cardiac fibrosis, improve mitochondrial oxidative stress and glucose metabolism.
Wei, 2025, Singapore (27)	HiPSC	β2-microglobulin knockdown	Male and female (C57BL/6 mice)/(MI/R)	Myocardial injection	CMs	Improve cardiac function, alleviate cardiac fibrosis, enhance the cell cycle activity of CMs.
Wang, 2025, China (28)	MSCs	Loaded with miR-222 + surface modificated with CTP+ encapsulated within hydrogel cardiac patches	Mice/(MI/R)	Pericardial space injection	CMs	Improve cardiac function, alleviate cardiac fibrosis and inflammation, inhibit cell apoptosis.
Wang, 2025, China (29)	MSCs	Loaded with CTLA-4 + surface modificated with mannose	C57BL/6 mice/MI	Tail vein injection	DCs	Improve cardiac function, alleviate cardiac fibrosis.
Wang, 2025, China (30)	MSCs	Surface modificated with IMTP	Male SD rat/(MI/R)	Tail vein injection	CMs, macrophages	Improve cardiac function, alleviate cardiac fibrosis and inflammation, promote angiogenesis.
Wang, 2025, China (31)	ADSCs	Loaded with SDF-1α+ surface modificated with anti-CD81 antibodies and cRGD	Male SD rat/MI	Tail vein injection	CMs, ECs, macrophages	Improve cardiac function, alleviate cardiac fibrosis and inflammation, promote angiogenesis, inhibit cell apoptosis.
Liu, 2025, China (32)	MSCs	Loaded with miR-222-3p	Male (C57BL/6J mice)/(MI/R)	Tail vein injection	CMs, ECs	Improve cardiac function, alleviate cardiac fibrosis and cell apoptosis, promote angiogenesis and mitochondrial metabolism.
Liu, 2025, China (33)	MSCs	Loaded with miR-181a-5p+ platelet membrane fusion	C57 mice/MI	Tail vein injection	CMs, macrophages	Improve cardiac function, alleviate cardiac fibrosis and inflammation, promote angiogenesis.
Li, 2025, China (34)	HEK 293T cells	Loaded with GPNMB mRNA+ encapsulated within hydrogels	Male (C57BL/6 mice)/MI	Myocardial injection	CMs, macrophages	Improve cardiac function, alleviate cardiac fibrosis and inflammation, inhibit cell apoptosis.
Chen, 2025, China (35)	Neutrophils	Loaded with PLGA+ neutrophil membrane fusion	Male (C57 mice)/(MI/R)	Tail vein injection	Neutrophils	Improve cardiac function, alleviate cardiac inflammation, promote angiogenesis.
Wang, 2024, China (36)	MSCs	Loaded with miR-223-3p	Male (C57BL/6J mice)/MI	Myocardial injection	CMs	Improve cardiac function, alleviate cardiac fibrosis and inflammation.
Mentkowski, 2024, America (37)	CDCs	Surface modificated with CMP	Male and female (C57BL/6J mice)/MI	Myocardial injection+ tail vein injection	CMs	Improve cardiac function, alleviate cardiac fibrosis and cell apoptosis.
Xue, 2024, China (38)	CMs，macrophages	Loaded with Mito Q+ macrophage membrane fusion	Male (C57BL/6 mice)/ (MI/R)	Pericardial space injection	CMs, macrophages	Improve cardiac function, alleviate cardiac fibrosis and inflammation, promote angiogenesis, inhibit cell apoptosis.
Zou, 2024, China (39)	H9c2 cells	Loaded with GDF-15	SD rat/MI	Myocardial injection	CMs	Improve cardiac function, alleviate cardiac fibrosis and inflammation, promote angiogenesis, inhibit cell apoptosis.
Zhang, 2024, China (40)	MSCs	Loaded with PLGF+ surface modificated with CHP	Male (C57BL/6J mice)/MI	Tail vein injection	CMs	Improve cardiac function, alleviate cardiac fibrosis and inflammation, promote angiogenesis.
Yin, 2024, China (41)	HEK 293F cells	WTAP knockdown+ surface modificated with CHP	Male (C57BL/6J mice)/(MI/R)	Tail vein injection	CMs	Improve cardiac function, alleviate cardiac fibrosis and cell apoptosis, improve mitochondrial oxidative stress.
Tan, 2024, China (42)	TSCs cells	Encapsulated within hyaluronic acid hydrogels	Male (C57BL/6J mice)/MI	Pericardial space injection	CMs	Improve cardiac function, alleviate cardiac fibrosis and inflammation, promote angiogenesis, inhibit cell apoptosis.
Lai, 2024, China (43)	MSCs	Loaded with high-affinity SIRPα variants + platelet membrane fusion	Male (C57BL/6J mice)/(MI/R)	Myocardial injection + tail vein injection	CMs, ECs, macrophages	Improve cardiac function, alleviate cardiac fibrosis and inflammation, inhibit cell apoptosis.
Gu, 2024, China (44)	MSCs	Loaded with miR-302 mimics+ surface modificated with CMP	Male (C57BL/6 mice)/ (MI/R)	Tail vein injection	CMs	Improve cardiac function, alleviate cardiac fibrosis and inflammation, inhibit cell apoptosis.
Das, 2024, India (45)	ADSCs	Encapsulated within gallic-acid-containing polyurethane scaffolds	Male SD rat/MI	Myocardial implantation	CMs, ECs	Improve cardiac function, alleviate cardiac fibrosis and inflammation, promote angiogenesis and mitochondrial oxidative stress.
Zhu, 2023, China (46)	MSCs	Loaded with miR-214-3p	Male SD rat/MI	Myocardial injection	CMs, ECs	Improve cardiac function, alleviate cardiac fibrosis and inflammation, promote angiogenesis, inhibit cell apoptosis.
Yu, 2023, China (47)	EPCs	Encapsulated within hydrogel microspheres+ pre-treatment with Silicate ion solution	C57BL/6 mice/MI	Myocardial injection	CMs, ECs	Improve cardiac function, alleviate cardiac fibrosis and cell apoptosis, promote angiogenesis.
Lin, 2023, China (48)	MSCs	Encapsulated within COL-I/TA-containing CS hydrogels	Male rat/MI	Myocardial injection	CMs, ECs, macrophages	Improve cardiac function, alleviate cardiac fibrosis and inflammation, inhibit cell apoptosis.
Lee, 2023, Korea (49)	ApoNVs	Surface modificated with dextran and CHP+ pre-treatment with STS	Male Fischer rat/ (MI/R)	Tail vein injection	CMs, macrophages	Improve cardiac function, alleviate cardiac fibrosis and inflammation, promote angiogenesis, inhibit cell apoptosis.
Huang, 2023, China (50)	Neutrophils	Loaded with CST+ surface modificated with MLC3 antibodies+ neutrophil membrane fusion	Male (C57BL/6 mice)/ (MI/R)	Tail vein injection	CMs, macrophages	Improve cardiac function, alleviate cardiac fibrosis and inflammation, inhibit cell apoptosis, promote mitochondrial metabolism.
Chen, 2023, China (51)	Macrophages	Loaded with Tβ4 + macrophage membrane fusion	Male (C57BL/6 mice)/MI	Tail vein injection	CMs, ECs	Improve cardiac function, alleviate cardiac fibrosis, promote angiogenesis.
Bheri, 2023, America (52)	CDCs	Loaded with miR-126	Male SD rat/ (MI/R)	Myocardial injection	CMs, ECs	Improve cardiac function, alleviate cardiac fibrosis, promote angiogenesis.
Zhong, 2023, China (53)	iPSC-CMs	Macrophage membrane fusion	Male (C57BL/6 mice)/MI	Tail vein injection	CMs	Improve cardiac function, alleviate cardiac fibrosis.
Zhang, 2022, China (54)	ADSCs	Loaded with melatonin	Male (C57BL/6J mice)/MI	Myocardial injection	CMs	Improve cardiac function, promote angiogenesis, Inhibit cell apoptosis.
Shiekh, 2022, India (55)	ADSCs	Encapsulated within PUAO-CPO-Collagen patches	Female SD rat/MI	Myocardial implantation	CMs, ECs	Improve cardiac function, alleviate cardiac fibrosis, promote angiogenesis and cardiac metabolism.
Monguió-Tortajada, 2022, Spain (56)	MSCs	Encapsulated within decellularised pericardial scaffolds filled with peptide hydrogels	Male and female (crossbreed pig)/MI	Myocardial implantation	CMs, macrophages	Improve cardiac function, alleviate cardiac fibrosis and inflammation.
Li, 2022, China (57)	Macrophages	Platelet membrane fusion	Male (C57BL/6 mice)/ (MI/R)	Tail vein injection	Macrophages	Improve cardiac function, alleviate cardiac fibrosis and inflammation.
Hu, 2022, China (58)	MSCs	Loaded with Islet-1 + encapsulated within Ang-1 hydrogels	Male (C57BL/6 mice)/MI	Myocardial injection	ECs	Improve cardiac function, alleviate cardiac inflammation, promote angiogenesis.
Hao, 2022, China (59)	MSCs	Encapsulated within patches composed of collagen gel and decellularized ECM	Male (C57/B6 mice)/MI	Myocardial implantation	ECs, macrophages	Improve cardiac function, alleviate cardiac fibrosis and inflammation, promote angiogenesis.
Bao, 2022, China (60)	Neutrophils	MSN^HAL^ membrane fusion+ pre-treatment with STS	Female SD rat/MI	Tail vein injection	ECs, macrophages	Improve cardiac function, alleviate cardiac fibrosis and inflammation, promote angiogenesis.
Yuan, 2022, China (61)	MSCs	Loaded with miR-204	C57BL/6J mice/ (MI/R)	Myocardial injection	CMs, macrophages	Improve cardiac function, alleviate cardiac fibrosis and inflammation.
Zhu, 2021, China (62)	MSCs	MIF overexpression or knockdown	Male SD rat/MI	Myocardial injection	CMs, ECs	Improve cardiac function, alleviate cardiac fibrosis and cell apoptosis, promote angiogenesis.
Wang, 2021, China (63)	MSCs	Loaded with HIF-1α+ encapsulated within RGD-biotin hydrogels	Male SD rat/MI	Myocardial injection	CMs, ECs	Improve cardiac function, alleviate cardiac fibrosis and inflammation, promote angiogenesis, inhibit cell apoptosis.
Monguió-Tortajada, 2021, Spain (64)	ADSCs	Encapsulated within decellularised pericardial scaffolds	Crossbreed pig/MI	Myocardial implantation	ECs, macrophages	Alleviate cardiac fibrosis and inflammation, promote angiogenesis.
Li, 2021, China (65)	MSC	Platelet membrane fusion	Male (C57BL/6 mice)/ (MI/R)	Tail vein injection	ECs	Improve cardiac function, alleviate cardiac fibrosis and cell apoptosis, promote angiogenesis.
Kwon, 2021, America (66)	CDCs	Loaded with βARKct peptide	Male (C57BL/6 mice)/MI	Myocardial injection	CMs	Improve cardiac function, alleviate cardiac fibrosis and inflammation, inhibit cell apoptosis.
Xuan, 2020, America (67)	MSCs	Notch1 overexpression or knockdown	C57/B6 mice/MI	Myocardial injection	ECs	Improve cardiac function, alleviate cardiac fibrosis and cell apoptosis, promote angiogenesis.
Wang, 2020, America (68)	MSCs	Loaded with miR-101a-3p	Male (C57BL/6 mice)/MI	Tail vein injection	CMs, macrophages	Improve cardiac function, alleviate cardiac fibrosis and inflammation.
Huang, 2020, China (69)	MSCs	Pre-treatment with ATV	Male SD rat/MI	Myocardial injection	CMs, ECs	Improve cardiac function, alleviate cardiac fibrosis and inflammation, promote angiogenesis, inhibit cell apoptosis.
Song, 2019, China (70)	HEK 293T cells	Loaded with miR-21	Male (C57BL/6 mice)/MI	Myocardial injection	CMs, ECs	Improve cardiac function, alleviate cardiac fibrosis and cell apoptosis, promote angiogenesis.

ADSCs, adipose tissue derived stem cells; Ang-1, angiogenin-1; ApoNVs, apoptotic bodies-mimetic nanovesicles derived from apoptotic fibroblasts; ATV, atorvastatin; βARKct, β-adrenergic receptor kinase 2 C-terminal tail; CDCs, cardiosphere-derived cells; CHP, ischemic heart homing peptide, CSTSMLKAC; CMP, cardiomyocytes specific peptide, WLSEAGPVVTVRALRGTGSW; CMs, cardiomyocytes; COL-I, collagen type 1; CPO, calcium peroxide; CS, a typical silicate bioceramic; CST, catestatin; CTLA-4, cytotoxic T-lymphocyte antigen 4; CTP, heart targeting peptide, CSTSMLKAC; Cx43, connexin 43; DCs, dendritic cells; ECM, extracellular matrix; ECs, endothelial cells; EPCs, endothelial progenitor cells; GDF-15, growth differentiation factor-15; GPNMB, glycoprotein non-metastatic melanoma protein B; HIF-1α, hypoxia-inducible factor-1α; HiPSC, human induced pluripotent stem cell; IL-10, interleukin-10; IMTP, ischemic myocardium targeting peptide, CSTSMLKAC; iPSC-CMs, cardiomyocytes induced by pluripotent stem cells; Islet-1, insulin gene enhancer protein ISL-1; MI, myocardial infarction; MIF, migration inhibitory factor; MI/R, myocardial ischemia/reperfusion; MiR, microRNA; Mito Q, a well-established mitochondrial-targeted antioxidant; MLC3, myosin light chain 3; MSCs, mesenchymal stem cells; NDUFS1, a core subunit of mitochondrial complex I; Notch1, Notch receptor 1; PLGA, poly lactic-co-glycolic acid; PLGF, placental growth factors; PUAO, antioxidant polyurethane; RGD, arginine-glycine-aspartate; SIRPα, signal regulatory protein α; SIRT3, sirtuin-3; STS, staurosporine; TA, tannic acid; Tβ4, thymosin β4; TSCs, trophoblast stem cells; VEGFA, Vascular Endothelial Growth Factor A; WTAP, Wilms' tumor 1-associating protein.

Modification methods for engineered EVs included internal loading or overexpression or knockdown of signal molecules (32/50), surface modification (12/50), membrane fusion (10/50), pre-treatment of EVs (5/50) and combined with biological materials such as gels, scaffolds, or patches (13/50). Study animals included Sprague-Dawley (SD) rats (15/50), Fischer rats (1/50), C57BL/6J mice (32/50), crossbreed pigs (2/50).

The main cardiovascular diseases included MI (33/50) and myocardial ischemia/reperfusion (MI/R) injury (17/50). Administration methods for engineered EVs involved tail vein injection (20/50), cardiac injection or implantation (25/50), intragastric injection (3/50) and tail vein injection combined with cardiac injection (2/50). Target cells of engineered EVs primarily included cardiomyocytes (CMs), macrophages, ECs, and smooth muscle cells (SMCs), etc. Engineered EVs demonstrated therapeutic effects by reducing inflammatory responses and oxidative stress, promoting angio-genesis, regulating apoptosis, improving fibrosis and metabolism.

### 3.3 Quality assessment of included studies

The quality of the 50 included animal studies was assessed using SYRCLE's Risk of Bias (ROB). This tool was adapted from the ROB tool developed by the Cochrane Collaboration for randomized controlled trials (RCTs), and was specifically designed for assessing the risk of bias in animal intervention studies. The tool consisted of 10 items, corresponding to 6 types of bias. For each item, the reviewer determined whether it was “yes” (low risk), “no” (high risk), or “unclear” (insufficient reporting) (20). The analysis revealed a low risk of bias in selective outcome reporting across all studies, while risks related to randomized outcome assessment and other sources of bias were generally unclear. Most studies demonstrated low risk concerning sequence generation (27/50), baseline characteristics (42/50), and incomplete outcome data (48/50). Moreover, risks related to allocation concealment (43/50), random housing (41/50), blinding of performance bias (42/50), and blinding of detection bias (44/50) were assessed as unclear. Detailed quality assessment results were provided in Table 2.

**Table 2:** Quality evaluation of the included studies according to SYRCLE's ROB tool.

**Author, year, country**	**Selection bias**			**Performance bias**		**Detection bias**		**Attrition bias**	**Reporting bias**	**Other sources of bias**
	**Sequence generation**	**Baseline characteristics**	**Allocation concealment**	**Random housing**	**Blinding**	**Random outcome assessment**	**Blinding**	**Incomplete outcome data**	**Selective outcome reporting**	**Other**
Mun, 2026, Korea (21)	Unclear	Low risk	Unclear	Unclear	Unclear	Unclear	Low risk	Low risk	Low risk	Unclear
Luo, 2026, China (22)	Low risk	Unclear	Unclear	Unclear	Unclear	Unclear	Unclear	Low risk	Low risk	Unclear
Liu, 2026, China (23)	Low risk	Unclear	Low risk	Unclear	Low risk	Unclear	Unclear	Low risk	Low risk	Unclear
Zhou, 2025, China (24)	Low risk	Unclear	Unclear	Unclear	Unclear	Unclear	Unclear	Low risk	Low risk	Unclear
Zhao, 2025, China (25)	Unclear	Low risk	Unclear	Unclear	Unclear	Unclear	Unclear	Low risk	Low risk	Unclear
Yang, 2025, China (26)	Low risk	Low risk	Unclear	Unclear	Unclear	Unclear	Unclear	Low risk	Low risk	Unclear
Wei, 2025, Singapore (27)	Unclear	Low risk	Unclear	Low risk	Unclear	Unclear	Unclear	Low risk	Low risk	Unclear
Wang, 2025, China (28)	Unclear	Low risk	Unclear	Unclear	Unclear	Unclear	Unclear	Low risk	Low risk	Unclear
Wang, 2025, China (29)	Low risk	Low risk	Unclear	Unclear	Low risk	Unclear	Unclear	Low risk	Low risk	Unclear
Wang, 2025, China (30)	Low risk	Low risk	Unclear	Unclear	Unclear	Unclear	Low risk	Low risk	Low risk	Unclear
Wang, 2025, China (31)	Low risk	Unclear	Low risk	Unclear	Unclear	Unclear	Unclear	Low risk	Low risk	Unclear
Liu, 2025, China (32)	Low risk	Unclear	Unclear	Unclear	Unclear	Unclear	Unclear	Low risk	Low risk	Unclear
Liu, 2025, China (33)	Unclear	Low risk	Unclear	Unclear	Unclear	Unclear	Unclear	Low risk	Low risk	Unclear
Li, 2025, China (34)	Unclear	Low risk	Unclear	Unclear	Unclear	Unclear	Unclear	Low risk	Low risk	Unclear
Chen, 2025, China (35)	Low risk	Low risk	Unclear	Unclear	Unclear	Unclear	Unclear	Low risk	Low risk	Unclear
Wang, 2024, China (36)	Unclear	Low risk	Unclear	Unclear	Unclear	Unclear	Unclear	Low risk	Low risk	Unclear
Mentkowski, 2024, America (37)	Unclear	Low risk	Unclear	Unclear	Unclear	Unclear	Unclear	Low risk	Low risk	Unclear
Xue, 2024, China (38)	Unclear	Low risk	Unclear	Unclear	Unclear	Unclear	Unclear	Low risk	Low risk	Unclear
Zou, 2024, China (39)	Unclear	Low risk	Unclear	Low risk	Unclear	Unclear	Unclear	Low risk	Low risk	Unclear
Zhang, 2024, China (40)	Unclear	Low risk	Unclear	Unclear	Unclear	Unclear	Unclear	Low risk	Low risk	Unclear
Yin, 2024, China (41)	Unclear	Low risk	Unclear	Low risk	Unclear	Unclear	Unclear	Low risk	Low risk	Unclear
Tan, 2024, China (42)	Unclear	Low risk	Unclear	Low risk	Unclear	Unclear	Unclear	Low risk	Low risk	Unclear
Lai, 2024, China (43)	Low risk	Low risk	Unclear	Low risk	Unclear	Unclear	Unclear	Low risk	Low risk	Unclear
Gu, 2024, China (44)	Low risk	Low risk	Unclear	Unclear	Unclear	Unclear	Unclear	Low risk	Low risk	Unclear
Das, 2024, India (45)	Low risk	Low risk	Unclear	Low risk	Unclear	Unclear	Unclear	Low risk	Low risk	Unclear
Zhu, 2023, China (46)	Unclear	Low risk	Low risk	Unclear	Low risk	Unclear	Unclear	Low risk	Low risk	Unclear
Yu, 2023, China (47)	Low risk	Low risk	Unclear	Low risk	Unclear	Unclear	Low risk	Low risk	Low risk	Unclear
Lin, 2023, China (48)	Unclear	Low risk	Unclear	Unclear	Unclear	Unclear	Unclear	Low risk	Low risk	Unclear
Lee, 2023, Korea (49)	Unclear	Low risk	Unclear	Unclear	Unclear	Unclear	Unclear	Low risk	Low risk	Unclear
Huang, 2023, China (50)	Low risk	Low risk	Unclear	Low risk	Unclear	Unclear	Unclear	Low risk	Low risk	Unclear
Chen, 2023, China (51)	Low risk	Low risk	Unclear	Unclear	Unclear	Unclear	Unclear	Low risk	Low risk	Unclear
Bheri, 2023, America (52)	Low risk	Low risk	Low risk	Unclear	Low risk	Unclear	Unclear	Low risk	Low risk	Unclear
Zhong, 2023, China (53)	Low risk	Unclear	Unclear	Unclear	Unclear	Unclear	Unclear	Low risk	Low risk	Unclear
Zhang, 2022, China (54)	Low risk	Unclear	Unclear	Unclear	Unclear	Unclear	Unclear	Low risk	Low risk	Unclear
Shiekh, 2022, India (55)	Unclear	Unclear	Unclear	Unclear	Unclear	Unclear	Unclear	Low risk	Low risk	Unclear
Monguió-Tortajada, 2022, Spain (56)	Low risk	Low risk	Low risk	Unclear	Low risk	Unclear	Low risk	Low risk	Low risk	Unclear
Li, 2022, China (57)	Low risk	Low risk	Unclear	Unclear	Unclear	Unclear	Unclear	Low risk	Low risk	Unclear
Hu, 2022, China (58)	Low risk	Low risk	Unclear	Unclear	Unclear	Unclear	Unclear	Low risk	Low risk	Unclear
Hao, 2022, China (59)	Low risk	Low risk	Unclear	Unclear	Unclear	Unclear	Unclear	Low risk	Low risk	Unclear
Bao, 2022, China (60)	Unclear	Low risk	Unclear	Unclear	Unclear	Unclear	Unclear	Low risk	Low risk	Unclear
Yuan, 2022, China (61)	Low risk	Low risk	Unclear	Low risk	Unclear	Unclear	Unclear	Low risk	Low risk	Unclear
Zhu, 2021, China (62)	Unclear	Low risk	Low risk	Unclear	Low risk	Unclear	Low risk	Low risk	Low risk	Unclear
Wang, 2021, China (63)	Unclear	Low risk	Unclear	Unclear	Unclear	Unclear	Unclear	Low risk	Low risk	Unclear
Monguió-Tortajada, 2021, Spain (64)	Low risk	Low risk	Unclear	Unclear	Unclear	Unclear	Unclear	Unclear	Low risk	Unclear
Li, 2021, China (65)	Low risk	Low risk	Unclear	Unclear	Unclear	Unclear	Unclear	Low risk	Low risk	Unclear
Kwon, 2021, America (66)	Unclear	Low risk	Unclear	Unclear	Unclear	Unclear	Unclear	Low risk	Low risk	Unclear
Xuan, 2020, America (67)	Unclear	Low risk	Unclear	Unclear	Unclear	Unclear	Unclear	Low risk	Low risk	Unclear
Wang, 2020, America (68)	Unclear	Low risk	Low risk	Unclear	Low risk	Unclear	Unclear	Low risk	Low risk	Unclear
Huang, 2020, China (69)	Low risk	Low risk	Unclear	Unclear	Low risk	Unclear	Low risk	Low risk	Low risk	Unclear
Song, 2019, China (70)	Low risk	Low risk	Unclear	Unclear	Unclear	Unclear	Unclear	Unclear	Low risk	Unclear

### 3.4 Outcome analysis

This systematic review included a total of 50 animal studies. We mainly summarized the results from 4 aspects: ① The modification, extraction and identification of engineered EVs. ② The targeted strategies and tracing experiments in animals. ③ The *in vivo* therapeutic effects of engineered EVs in animals with IHD. ④ Revealing the intrinsic relationship and matching rules between the multi-modal therapy effects and the modification methods.

#### 3.4.1 Modification, extraction and identification of engineered EVs

##### 3.4.1.1 Modification methods of engineered EVs

After systematically analyzing the detailed processes of artificial modification of engineered EVs across 50 studies, we summarized the modification methods into 5 types: loading/knockdown of signaling molecules inside EVs, modification of the EVs' membrane surface, fusion of the EVs' membranes, encapsulation of the EVs in biotechnological materials, and pre-treatment of the EVs. Among the 50 studies, 32 modified the EVs by loading/ knockdown the signaling molecules using genetic engineering techniques, such as lentiviral transduction, siRNA transfection, liposome transduction, CRISPR/Cas9 gene editing and molecular cloning; and physical or chemical methods, such as electroporation, ultrasound treatment, chitosan (CS) electrostatic adsorption, compression and hydrophobic interactions. Membrane surface modification was applied in 12 studies, involving covalent binding, click chemistry, sequence fusion, lentiviral transduction and emulsion extrusion, to modify the membrane surface of the parental cells or EVs. There were 10 studies reporting membrane fusion phenomena, which were achieved through ultrasound treatment or compression, fusing the EVs membrane with platelet membrane, macrophage membrane, neutrophil membrane or 6-aminocaproic acid hydrochloride (HAL) membrane. Another 13 studies encapsulated the engineered exosomes in biotechnological materials, such as hyaluronic acid hydrogel, fibrin gel, CS hydrogel, collagen hydrogel, lysine-glycine-aspartic acid (RGD)-biotin hydrogel, antioxidant polyurethane scaffold, decellularized pericardial scaffold, decellularized polyurethane scaffold or decellularized heart patch. Finally, 5 studies pre-treated the EVs, including ginsenoside Rg1, staurosporine (STS), atorvastatin (ATV) or silicate ion solution to stimulate or co-culture primary cells, induce cell apoptosis, and increase the protein secretion of EVs. Specific details could be found in Table 3.

**Table 3:** Modification methods and identification of engineered EVs.

**Author, year, country**	**Parent cells of EVs**	**Interior loading/knockdown**	**Surface modification**	**Membrane fusion**	**Combined with biological materials**	**Pre-treatment**	**Extraction methods of EVs**	**Identification methods of EVs**
Mun, 2026, Korea (21)	MSCs	Polycarbonate membrane filters/IL-10 mRNA overexpression	Click chemistry/T peptide, anti-CD63 and MLC3 antibodies				Extrusion technology	TEM, NTA, DLS
Luo, 2026, China (22)	HEK 293T cells	Lentiviral transduction/Ndufs1 and VegfA mRNA overexpression	Lentiviral transduction/IMTP and Cx43 overexpression				Differential UC	TEM, NTA, WB (CD9, CD81, GM130)
Liu, 2026, China (23)	ADSCs, Macrophages			Extrusion technology/ cholesterol-modified macrophage membrane fusion			Extrusion technology	AFM, NTA, DLS, WB (CD68, CD90, GAPDH)
Zhou, 2025, China (24)	Gouqi				Methods for exosome-loaded fibrin/ encapsulated within fibrin gels		Density gradient centrifugation	TEM, NTA
Zhao, 2025, China (25)	MSCs					Co-incubation/ ginsenoside Rg1 pre-treatment	Extrusion technology, differential UC	TEM, NTA, WB (CD9, CD63, Alix, Calnexin)
Yang, 2025, China (26)	MSCs	Molecular cloning techniques and Lipofectamine TM 2000 transfection/SIRT3 and Insulin mRNA overexpression					Differential UC	TEM, NTA, ZetaView, WB (CD9, CD63, CD81, TSG101)
Wei, 2025, Singapore (27)	HiPSC	CRISPR/Cas9 gene editing technology/β2-microglobulin knockdown					Differential UC	TEM, NTA, ZetaView
Wang, 2025, China (28)	MSCs	Electroporation/miR-222 overexpression	Click chemistry/ surface modification with CTP		Photosensitive curing and force-sensing mechanotransduction/ encapsulated within hydrogel cardiac patches		UC	TEM, NTA, WB (CD63, CD9, TSG101, Alix)
Wang, 2025, China (29)	MSCs	Lentiviral transduction/CTLA-4 overexpression	NR/surface modification with mannose				UC	TEM, ZetaView, WB (CD63, CD81)
Wang, 2025, China (30)	MSCs		Lentiviral transduction/IMTP overexpression				UC	TEM, NTA, WB (CD63, CD9)
Wang, 2025, China (31)	ADSCs	Lentiviral transduction/SDF-1α overexpression	Film hydration method/surface modification with anti-CD81 antibodies and cRGD				UC	TEM, NTA, WB (CD81, TSG101)
Liu, 2025, China (32)	MSCs	Mimic transduction/miR-222-3p overexpression					Extrusion technology	TEM, NTA, DLS, WB (CD81, CD9, TSG101, Alix)
Liu, 2025, China (33)	MSCs	Lipofectamine TM 2000 transfection/miR-181a-5p overexpression		Extrusion technology/platelet membrane fusion			Extrusion technology	TEM, NTA, WB (Alix, P-selectin, β-actin)
Li, 2025, China (34)	HEK 293T cells	Electroporation and ultrafiltration/loaded with LFA-1 mRNA			Solution mixing and cross-linking/ preparation of hydrogels		Differential UC, filtration	TEM, NTA
Chen, 2025, China (35)	Neutrophils	Nanoprecipitation method/loaded with PLGA		Ultracentrifugation and nanoprecipitation method/neutrophil membrane fusion			UC	DLS, WB (TNF-αR, IL-6R)
Wang, 2024, China (36)	MSCs	Exo-Fect Exosome Transfection Kit/loaded with miR-223-3p					Differential UC	TEM, NTA, WB (CD63, CD9, CD81, Alix)
Mentkowski, 2024, America (37)	CDCs		Lentiviral transduction/surface modification with CMP				Ultrafiltration, SEC	NTA, cryo-TEM, WB (Alix, CD81, HSP90, TSG101)
Xue, 2024, China (38)	CMs, macrophages	Extrusion technology/ loaded with Mito Q		Extrusion technology/macrophage membrane fusion			Extrusion technology, UC	TEM, NTA, ZetaView, WB (Cx43, α-actin, CD163, CD206)
Zou, 2024, China (39)	H9c2 cells	Lentiviral transduction/GPNMB overexpression					Differential UC	TEM, NTA, WB (CD63, CD81, TSG101)
Zhang, 2024, China (40)	MSCs	Ultrasonic treatment/ loaded with PLGF	Covalent binding/ surface modification with CHP				TFF, Differential UC	TEM, NTA, WB
Yin, 2024, China (41)	HEK 293F cells	Electroporation and siRNA transfection/ WTAP knockdown	Kit/surface modification with CHP				EVs were purchased directly	TEM, NTA, WB (TSG101, CD81, GM130)
Tan, 2024, China (42)	TSCs cells				Bioengineering technology/encapsulated within hyaluronic acid hydrogels		UC	TEM, NTA, WB (CD9, TSG101, calnexin)
Lai, 2024, China (43)	MSCs	Lentiviral transduction/ high-affinity SIRPα variants overexpression		Extrusion technology/platelet membrane fusion			UC, extrusion technology	TEM, NTA, WB (GPVI, VCAM1, SαV)
Gu, 2024, China (44)	MSCs	Electroporation/loaded with miR-302mimics	Click chemistry/ surface modification with CMP				Total EVs isolation reagent kit (Invitrogen)	TEM, NTA, flow cytometry (CD29, CD44, CD45, CD73)
Das, 2024, India (45)	ADSCs				Adsorption/encapsulated within gallic-acid-containing polyurethane scaffolds		Ultrafiltration	SEM, TEM, DLS, AFM, NTA, WB (CD9, CD81)
Zhu, 2023, China (46)	MSCs	Lentiviral transduction/miR-214-3p overexpression					EVs isolation reagent, centrifugation	TEM, NTA, WB (CD81, CD63, TSG101)
Yu, 2023, China (47)	EPCs				Microfluidic technology/encapsulated within hydrogel microspheres	Stimulated with Silicate ion solution/ enhanced EVs production	UC	TEM, NTA, WB (Alix, CD81, CD63)
Lin, 2023, China (48)	MSCs				NR/encapsulated within COL-I/TA-containing CS hydrogels		Differential centrifugation	NR
Lee, 2023, Korea (49)	ApoNVs		Co-incubation/ surface modification with dextran and CHP			Co-incubation with STS/ induced cell apoptosis	UC, extrusion technology	TEM, DLS
Huang, 2023, China (50)	Neutrophils	Emulsion extrusion technology/loaded with CST	Emulsion extrusion technology/surface modification with MLC3 antibodies	Emulsion extrusion technology/neutrophil membrane fusion			Emulsion-extrusion method	TEM, DLS
Chen, 2023, China (51)	Macrophages	Seal method/loaded with Tβ4		Kit and extrusion technology/macrophage membrane fusion			EVs extraction reagent kit	TEM, DLS, WB (CCR2, TSG101, Alix, CD9, CD63, Syntenin)
Bheri, 2023, America (52)	CDCs	Electroporation/loaded with miR-126					Differential UC	TEM, NTA, DLS
Zhong, 2023, China (53)	iPSC-CMs			Extrusion technology/Macrophage membrane fusion			UC	TEM, NTA, WB (CD9, CD63, CD81, CCR2)
Zhang, 2022, China (54)	ADSCs	Ultrasonic treatment and ultrafiltration/ loaded with melatonin					Extrusion technology	TEM, NTA, WB (TSG101, CD9, CD63, CD81, HSP70, calnexin)
Shiekh, 2022, India (55)	ADSCs				Solution addition and adsorption/encapsulated within PUAO-CPO-Collagen patch		NR	TEM, DLS, WB (CD9)
Monguió-Tortajada, 2022, Spain (56)	MSCs				Solution mixing/ encapsulated within decellularised pericardial scaffolds filled with peptide hydrogels		UC	NTA, cryo-TEM, flow cytometry (CD63, CD44)
Li, 2022, China (57)	Macrophages			Ultrasonic treatment and extrusion technology/platelet membrane fusion			UC	TEM, NTA, DLS, WB (TSG101, CD63, Alix, CD42c, GPIbα, CD62P)
Hu, 2022, China (58)	MSCs	Lentiviral transduction/Islet-1 overexpression			Solution mixing/encapsulated within Ang-1 hydrogels		UC	TEM, NTA, WB (Alix, CD63, CD9)
Hao, 2022, China (59)	MSCs				NR/encapsulated within patches composed of collagen gel and decellularized ECM		UC	TEM, WB (CD9, TSG101, calnexin)
Bao, 2022, China (60)	Neutrophils			Extrusion technology/MSN^HAL^ membrane fusion		Co-incubation with STS/induced cell apoptosis	Centrifugation	SEM, DLS, WB (LFA-1, C1q, integrin-β3, CD11b, CD45)
Yuan, 2022, China (61)	MSCs	Lipofectamine TM 2000 transfection/miR-204 overexpression					EVs extraction reagent kit, UC	TEM, NTA, WB (CD81, CD63, TSG101, calnexin)
Zhu, 2021, China (62)	MSCs	Lentiviral transduction/MIF overexpression or knockdown					EVs isolation reagent, centrifugation	TEM, NTA, WB (TSG101, CD63, CD81)
Wang, 2021, China (63)	MSCs	Lentiviral transduction/HIF-1α overexpression			NR/encapsulated within RGD-biotin hydrogels		Differential UC	TEM, NTA
Monguió-Tortajada, 2021, Spain (64)	ADSCs				NR/encapsulated within decellularised pericardial scaffolds		UC, SEC	Cryo-TEM, NTA, flow cytometry (CD9, CD63, CD81, CD29, CD44)
Li, 2021, China (65)	MSC			Extrusion technology/platelet membrane fusion			UC, multigelation	TEM, NTA, immunogold TEM, WB (CD9, Alix, TSG101, CD90)
Kwon, 2021, America (66)	CDCs	Lentiviral transduction/βARKct peptide overexpression					Differential UC	Electron microscope, Nanosight technology, WB (HSP70, flotillin)
Xuan, 2020, America (67)	MSCs	Adenovirus transfection/Notch1 overexpression or knockdown					SEC	TRPS, TEM, WB (TSG101, CD63, calnexin)
Wang, 2020, America (68)	MSCs	Electroporation/ miR-101a-3p overexpression					Differential centrifugation	TEM, NTA, DLS, WB (CD9, CD63)
Huang, 2020, China (69)	MSCs					Co-incubation/ ATV pre-treatment	Differential centrifugation	TEM, NTA, WB (Alix, TSG101, CD81, CD63)
Song, 2019, China (70)	HEK 293T cells	Vector transfection/ loaded with miR-21					UC	TEM, NTA, WB (CD9, CD63, CD81)

ADSCs, adipose tissue derived stem cells; Ang-1, angiogenin-1; ApoNVs, apoptotic bodies-mimetic nanovesicles derived from apoptotic fibroblasts; ATV, atorvastatin; βARKct, β-adrenergic receptor kinase 2 C-terminal tail; CCR2, C-C motif chemokine receptor 2; CDCs, cardiosphere-derived cells; CHP, ischemic heart homing peptide, CSTSMLKAC; CMP, cardiomyocytes specific peptide, WLSEAGPVVTVRALRGTGSW; CMs, cardiomyocytes; COL-I, collagen type 1; CPO, calcium peroxide; CS, a typical silicate bioceramic; CST, catestatin; CTLA-4, cytotoxic T-lymphocyte antigen 4; CTP, heart targeting peptide, CSTSMLKAC; Cx43, connexin 43; DLS, dynamic light scattering; ECM, extracellular matrix; EPCs, endothelial progenitor cells; EVs, extracellular vesicles; GDF-15, growth differentiation factor-15; GM130, golgi matrix protein 130; GPIbα, glycoprotein Ib alpha; GPNMB, glycoprotein non-metastatic melanoma protein B; GPVI, glycoprotein VI; HIF-1α, hypoxia-inducible factor-1α; HiPSC, human induced pluripotent stem cell; HSP70, heat shock protein 70; HSP90, heat shock protein 90; IL-6R, interleukin 6 receptor; IL-10, interleukin-10; IMTP, ischemic myocardium targeting peptide, CSTSMLKAC; iPSC-CMs, cardiomyocytes induced by pluripotent stem cells; Islet-1, insulin gene enhancer protein ISL-1; LFA-1, lymphocyte function-associated antigen 1; MIF, migration inhibitory factor; MiR, microRNA; Mito Q, a well-established mitochondrial-targeted antioxidant; MLC3, myosin light chain 3; MSCs, mesenchymal stem cells; NR, not reported; NDUFS1, a core subunit of mitochondrial complex I; Notch1, Notch receptor 1; NTA, nanoparticle tracking analysis; PLGA, poly lactic-co-glycolic acid; PLGF, placental growth factors; PUAO, antioxidant polyurethane; RGD, arginine-glycine-aspartate; SαV, signal regulatory protein α variants; SDF-1α, stromal cell-derived factor-1 alpha; SEC, size exclusion chromatography; SIRPα, signal regulatory protein α; SIRT3, sirtuin-3; STS, staurosporine; TA, tannic acid; Tβ4, thymosin β4; TEM, transmission electron microscopy; TFF, tangential flow filtration; TNF-αR, tumor necrosis factor-alpha receptor; TSCs, trophoblast stem cells; TSG101, tumor susceptibility 101; UC, ultracentrifugation; VCAM1, vascular cell adhesion molecule 1; VEGFA, vascular endothelial growth factor A; WB, western blot; WTAP, Wilms' tumor 1-associating protein.

##### 3.4.1.2 Extraction and identification of engineered EVs

As shown in Table 3, the 50 included studies mainly used differential ultracentrifugation, ultrafiltration, extrusion techniques, size exclusion chromatography or kits to extract and separate EVs. Methods such as transmission electron microscopy (TEM), nanoparticle tracking analysis (NTA), dynamic light scattering (DLS), nanoplasmonic flow cytometry, and Western blotting (WB) were used for the identification of EVs. The detection indicators of WB were mostly classic markers of EVs (such as: CD9, CD63, CD81, TSG101, Alix, GM130, Calnexin) and specific proteins contained in EVs after engineering modification (such as: CD68, CD90, P-selectin, TNF-αR, IL-6R, HSP70, HSP90, Cx43, α-actin, CD163, CD206, GPVI, VCAM1, SαV and CCR2, etc).

#### 3.4.2 Targeting strategies and tracing experiments of engineered EVs in animals with IHD

##### 3.4.2.1 Methods of animal administration of engineered EVs

As shown in Table 4, in this systematic review, tail vein injection was the most commonly used administration method for delivering engineered EVs to the animal bodies. 21 studies employed the tail vein injection delivery method, including 16 mouse studies and 5 rat studies. At the same time, *in situ* cardiac delivery was also a relatively common administration method, including *in situ* cardiac injection, pericardial space injection, and the implantation of biological engineering materials such as gels, scaffolds, or patches into the heart. 29 studies adopted the *in situ* cardiac delivery method, including 16 mouse studies, 11 rat studies, and 2 hybrid Landrace pig studies. Thus, the tail vein injection route was more commonly used for drug administration in mouse models, while *in situ* cardiac delivery was the main method in rat and pig models. Compared with direct cardiac injection, tail vein injection in mice had a higher animal survival rate and requires a lower amount of EVs, making it more economical and efficient. Given that rats and hybrid Landrace pigs had larger body sizes and heart volumes, *in situ* cardiac delivery not only has higher operational feasibility but also achieves higher drug absorption rate and utilization rate.

**Table 4:** *In vivo* targeting strategies and tracing experiments of engineered EVs.

**Author, year, country**	**Animal strain**	**Methods and dosage of animal administration of EVs**	**Intervention period of EVs**	**Targeted strategies of engineering EVs**	**Labeling and tracing methods of engineering EVs**	**Tracer experiments results of engineering EVs**
Mun, 2026, Korea (21)	Mice	Tail vein injection/ (300 μg/time, once)	1 week	Surface modificated with T peptide and anti-MLC3 antibody	Cy5.5/IVIS imaging system	Liver, Heart > Lung = Spleen = Kidney (4 h).
Luo, 2026, China (22)	Mice	Tail vein injection/ (200 μg/time,7 times)	4 weeks	Surface modificated with IMTP	DiR/optical Imaging System	Liver > Heart > Kidney = Lung = Spleen (24 h). The IMTP modification and the overexpression of Cx43 enhanced the targeting of EVs to the infarcted myocardium.
Liu, 2026, China (23)	Rat	Tail vein injection/ (5 × 10^7^ EVs/time, once)	4 weeks	Macrophage membrane fusion	DiD/IVIS imaging system	Liver > Heart > Kidney > Lung = Spleen (24 h). Hy-gNVs@Chol gradually accumulated in the infarcted myocardium, reaching the maximum cardiac fluorescence at 24 h, and remained clearly visible after 72 h. It had an effective delivery capability for ischemic myocardial tissue.
Zhou, 2025, China (24)	Mice	Myocardial injection/ (1 × 10^8^EVs/time, once)	2 weeks	Myocardial injection	DiR/LAGO X optical Imaging System	The GqDNVs released by the GqDNVs Gel rapidly spread throughout the Heart starting from 2 h after administration; compared with cardiac infusion or injection of GqDNVs, the GqDNVs-Gel intervention could cause GqDNVs to remain highly retained in the heart for 12 h after administration and persist until 24 h.
Zhao, 2025, China (25)	Rat	Myocardial injection/ (NR, once)	1 day	Myocardial injection	DiL/NR	The H9c2 and AC16 cells can internalize DiL-labeled ACDV, and this process lasts for 6 h.
Yang, 2025, China (26)	Rat	Myocardial injection/ (1 × 10^11^EVs/kg body weight, once)	4 weeks	Myocardial injection	DiD/IVIS imaging system	12 h after injection, the myocardial uptake in the Exo-I-S group was significantly higher than that in the Exo-WT group, and the clearance rate of the Exo-WT group was faster than that of the Exo-I-S group.
Wei, 2025, Singapore (27)	Mice	Myocardial injection/ (2 × 10^10^EVs/time, once)	4 weeks	Myocardial injection	PKH67/fluorescence microscope	When B2MKO hiPSC-NVs were co-cultured with hiPSC-CMs, green fluorescence began to appear 4 h after incubation and persisted for 4 days.
Wang, 2025, China (28)	Mice	Pericardial space injection/ (NR, once)	3 weeks	Surface modificated with CTP + pericardial space injection	DiD/IVIS imaging system	Liver > Heart > Brain = Kidney = Lung = Spleen (24 h). The total radiation efficiency of the DiD-labeled fluorescence in the CTP-targeted group was twice that of the non-targeted group. The addition of the injectable hydrogel did not alter the *in vivo* targeting delivery capability of TeEvs.
Wang, 2025, China (29)	Mice	Tail vein injection/ (300μg/time,4 times)	4 weeks	NR	DiR/IVIS imaging system	Liver > Spleen > Kidney > Heart (48 h).
Wang, 2025, China (30)	Rat	Tail vein injection/ (400μg/time, once)	4 weeks	Surface modificated with IMTP	DiR/IVIS imaging system	The border area of myocardial infarction = The normal area of myocardial non-infarction > Liver > Kidney > Spleen > Lung (48 h).
Wang, 2025, China (31)	Rat	Tail vein injection/ (1 × 10^7^EVs/time, once)	3 weeks	NR	DiR/IVIS imaging system	In the TNB group, stable fluorescence was detected in the cardiac area 2 h after injection, reaching its peak at 48 h and persisting until 72 h. In contrast, the NB group only showed fluorescence in the abdomen 2 h after injection. The number of exosomes absorbed in the MI area by the TNB group was significantly higher than that of the NB group.
Liu, 2025, China (32)	Mice	Tail vein injection/ (NR, once)	2 weeks	NR	NR	NR
Liu, 2025, China (33)	Mice	Tail vein injection/ (5 × 10^9^EVs/time, once)	4 weeks	Platelet membrane fusion	DiR/PerkinElmer vivo imaging	Liver > Heart = Lung = Spleen = Kidney (1day,3days,7days). P-181-NV effectively reduced the retention rate of the liver and significantly increased the retention rate of the heart.
Li, 2025, China (34)	Mice	Myocardial injection/ (NR, once)	2 weeks	Myocardial injection	DiR/IVIS imaging system	Heart > Liver = Lung (3days,7days). The dECM/GP hydrogel reduced the clearance of EVS@GPNMB and prolonged their retention time in the body.
Chen, 2025, China (35)	Mice	Tail vein injection/ (200μL/time, once)	4 weeks	Neutrophil membrane fusion	DiD/IVIS imaging system	The fluorescence intensity in the heart infarction area of the Neu-NP group significantly increased 3 h after administration, indicating that Neu-NP could recognize chemotactic signals and migrate to the heart MI/R injury area.
Wang, 2024, China (36)	Mice	Myocardial injection/ (5μg/time, once)	3 weeks	Myocardial injection	NR	NR
Mentkowski, 2024, America (37)	Mice	Myocardial and tail vein injection/ (100μL/time, once)	4 weeks	Surface modificated with CMP + myocardial injection	DiR or DiD/IVIS imaging system	Liver > Lung > Spleen > Intestine > Heart = Kidney = Brain = Bone (2 h,24 h). After intravenous injection throughout the body, CMP-EVs demonstrated greater cardiac targeting and increased cardiac retention after being administered intramyocardially.
Xue, 2024, China (38)	Mice	Pericardial space injection/ (NR, once)	2 weeks	Macrophage membrane fusion+ pericardial space injection	DiD/*in vivo* imaging	Mito Q@MMNv could remain in the body for more than 72 h, while traditional vesicles were quickly cleared. Compared with traditional vesicles, Mito Q@MMNv had a higher absorption efficiency in cardiac muscle cells and macrophages, and a longer retention time in cardiac tissue.
Zou, 2024, China (39)	Rat	Myocardial injection/ (100μg/time, once)	4 weeks	Myocardial injection	Dil/NR	NR
Zhang, 2024, China (40)	Mice	Tail vein injection/ (100μg/20 g body weight, once)	4 weeks	Surface modificated with CHP	DiR/IVIS imaging system	Heart > Liver = Spleen (24 h). CHP significantly increased the accumulation of EVs in the heart.
Yin, 2024, China (41)	Mice	Tail vein injection/ (200μg/time, once)	1 day	Surface modificated with CHP	DiR or DiL/*in vivo* imaging	Heart > Liver = Spleen = Lung (4 h). Loading EVs with targeted peptides was a promising strategy for targeted drug delivery.
Tan, 2024, China (42)	Mice	Pericardial space injection/ (15μL/time, once)	3 weeks	Pericardial space injection	DiO or DiR/IVIS imaging system	Intraperitoneal injection of EV-HA had good local sustained-release properties in mice.
Lai, 2024, China (43)	Mice	Myocardial injection/ (20μg/time, once) +tail vein injection/ (200μg/time,3 times)	3 weeks	Platelet membrane fusion + myocardial injection	DiD/IVIS imaging system	The EVs group showed higher abundance in the damaged heart, while other organs such as the liver, spleen, and lung showed lower fluorescence intensity, demonstrating the ability of EVs targeting the damaged heart.
Gu, 2024, China (44)	Mice	Tail vein injection/ (0.25μg/100 μL,14 times)	4 weeks	Surface modificated with CHP	DiL/NR	NR
Das, 2024, India (45)	Rat	Myocardial implantation/ (100μg/time, once)	8 weeks	Myocardial implantation	Calcein AM/NR	NR
Zhu, 2023, China (46)	Rat	Myocardial injection/ (50μg/time, once)	4 weeks	Myocardial injection	DiL/Laser-scanning confocal microscope	After 6 h of injection, miR-214OE-EVs was effectively presented in cardiomyocytes and ECs of the infarcted heart.
Yu, 2023, China (47)	Mice	Myocardial injection/ (20μg/time, once)	3 weeks	Myocardial injection	PKH26/NR	NR
Lin, 2023, China (48)	Rat	Myocardial injection/ (NR, once)	4 weeks	Myocardial injection	NR/NR	NR
Lee, 2023, Korea (49)	Rat	Tail vein injection/ (50μg/time,2 times)	4 weeks	Surface modificated with CHP	Cy5.5/PerkinElmer vivo imaging	Liver > Kidney > Heart > Lung > Spleen (24 h). CHP-ApoEV was superior to nEV and ApoEV in the accumulation of IR-damaged heart, indicating that targeted CHP-ApoEV achieved effective and safe delivery after systemic administration.
Huang, 2023, China (50)	Mice	Tail vein injection/ (NR, once)	4 weeks	Surface modificated with MLC3 antibodies + neutrophil membrane fusion	DiD/*in vivo* imaging	Liver, Spleen, Heart, Lung, Kidneys (24/72 h). External modification of neutrophil membranes and MLC3 antibodies endowed CMEV with the ability to target the core of myocardial lesions in a graded way.
Chen, 2023, China (51)	Mice	Tail vein injection/ (200μg/time,3 times)	4 weeks	Macrophage membrane fusion	PKH26/IVIS imaging system	Heart, Liver, Lung, Spleen, Kidney (1/3/6/12/24/72 h). Membrane decoration enhanced EVs aggregation in the target organ.
Bheri, 2023, America (52)	Rat	Myocardial injection/ (5 or 10μg/kg body weight, once)	4 weeks	Myocardial injection	DiR/IVIS imaging system	At 7 days of injection, there was no significant difference in retention between the EV and miR-126-EV groups, and there was significant retention in the myocardium of both groups compared to the control group.
Zhong, 2023, China (53)	Mice	Tail vein injection/ (1 × 10^11^ EVs/time, once)	4 weeks	Macrophage membrane fusion	NR/IVIS imaging system	Heart, liver, spleen, lung and kidneys (3 days). After 6 h of injection, MMM-EVs accumulated more in the injured heart compared with EVs and had the ability to target the infarcted heart.
Zhang, 2022, China (54)	Mice	Myocardial injection/ (20μL/time, once)	4 weeks	Myocardial injection	PKH26/NR	NR
Shiekh, 2022, India (55)	Rat	Myocardial implantation/ (NR, once)	8 weeks	Myocardial implantation	PKH26 or PKH67/confocal microscopy	Sustained EVs release was observed with about 80% of exosomes released within 8 days.
Monguió-Tortajada, 2022, Spain (56)	Pig	Myocardial implantation/ (NR, once)	4 weeks	Myocardial implantation	NIR815/infrared imaging system	The fluorescence signal in scaffolds and fibrotic scar tissue was significantly increased.
Li, 2022, China (57)	Mice	Tail vein injection/ (200μg/time, once)	4 weeks	Platelet membrane fusion	DID/IVIS imaging system	Heart > Liver = Spleen = Lung = Kidneys = Brain (3 h).PM-EV preferentially accumulate in ischemic myocardium.
Hu, 2022, China (58)	Mice	Myocardial injection/ (20μg/time, once)	4 weeks	Myocardial injection	PKH26/NR	NR
Hao, 2022, China (59)	Mice	Myocardial implantation/ (NR, once)	4 weeks	Myocardial implantation	DiL/IVIS imaging system	The encapsulation of EVs in collagen gel enhanced the retention of EVs in the heart and the sustainable release of EVs in the gel.
Bao, 2022, China (60)	Rat	Tail vein injection/ (2 mg/kg body weight, once)	4 weeks	NR	DiR/IVIS imaging system	Lung > Heart > Liver > Spleen and Kidney (3 h). eNAB^HAL^ could accumulate significantly in the heart infarction area, and the accumulation reached a peak at 12 h after injection and gradually decreased at 24 h.
Yuan, 2022, China (61)	Mice	Myocardial injection/ (25μL/time, once)	3 days	Myocardial injection	NR/NR	NR
Zhu, 2021, China (62)	Rat	Myocardial injection/ (50μg/time, once)	4 weeks	Myocardial injection	DiL/immunofluorescence	6 h after the MI model was established and vesicles were injected, a large area of DiL-labeled EVs in the myocardium and near the endothelium were positive.
Wang, 2021, China (63)	Rat	Myocardial injection/ (50μg/time, once)	4 weeks	Myocardial injection	DiR/IVIS imaging system	EVs accumulated significantly in the myocardium and EV-RGD hydrogels increased the retention and stability of EVs.
Monguió-Tortajada, 2021, Spain (64)	Pig	Myocardial implantation/ (2 × 10^7^ EVs/time, once)	1 week	Myocardial implantation	NIR815/near-infrared fluorescence scanning	6 days after implantation, labeled EVs were detected in both the graft and the infarct core and persisted only in the damaged area of the heart in the treated animals.
Li, 2021, China (65)	Mice	Tail vein injection/ (100μg/time,4 times)	4 weeks	Platelet membrane fusion	DiD or DiO/IVIS imaging system	Liver > Heart > Spleen > Lung > Kidney and Brain (24 h). PM-EV mainly accumulated in the anterior part of the left ventricle, especially in the area of ischemic necrosis and in the area of platelet infiltration after ischemia.
Kwon, 2021, America (66)	Mice	Myocardial injection/ (3 × 10^8^ EVs/time, once)	4 weeks	Myocardial injection	NR/NR	NR
Xuan, 2020, America (67)	Mice	Myocardial injection/ (20μL/time, once)	4 weeks	Myocardial injection	PKH26/NR	NR
Wang, 2020, America (68)	Mice	Tail vein injection/ (2 mg/kg body weight/time,2 times)	2 weeks	NR	DiD/IVIS imaging system	Spleen > Liver > Lung > Bone marrow > Heart > Kidney > Brain (5 h). EVs could accumulate in the infarcted heart.
Huang, 2020, China (69)	Rat	Myocardial injection/ (10μg/time, once)	4 weeks	Myocardial injection	PKH26/immunofluorescence	PKH26-labeled EVs accumulated significantly around ECs and fibroblasts in the infarcted heart.
Song, 2019, China (70)	Mice	Myocardial injection/ (20μg/time, once)	4 weeks	Myocardial injection	PKH26/immunofluorescence	MiR-21-EV could be effectively distributed into CMs and ECs of the infarcted heart.

ACDV, artificial cell derived vesicle; ApoEV, apoptotic bodies-mimetic nanovesicles derived from apoptotic fibroblasts; chol, cholesterol; CHP, ischemic heart homing peptide, CSTSMLKAC; CMEV, cellular membrane-engineered nanovesicle; CMP, cardiomyocytes specific peptide, WLSEAGPVVTVRALRGTGSW; CMs, cardiomyocytes; CTP, heart targeting peptide, CSTSMLKAC; Cx43, connexin 43; ECM, extracellular matrix; ECs, endothelial cells; eNABs, engineered neutrophil apoptotic bodies; EV, extracellular vesicle; GqDNVs, loaded Gouqi-derived nanovesicles; iPSC-CMs, cardiomyocytes induced by pluripotent stem cells; HA, hyaluronic acid; HAL, hexyl 5-aminolevulinate hydrochloride; Hy-gNVs, hybrid gelled nanovesicles; IMTP, ischemic myocardium targeting peptide, CSTSMLKAC; MI, myocardial infarction; MI/R, myocardial ischemia/reperfusion; miR-214OE, microRNA-214 overexpression; Mito Q, a well-established mitochondrial-targeted antioxidant; MLC3, myosin light chain 3; MMM, mononuclear macrophage membrane; NB, blank nanobubble; NR, not reported; Neu-NP, neutrophil membrane-coated poly lactic-co-glycolic acid nanoparticle; NV, nanovesicle; PM-EV, platelet membrane modified EV; RGD, arginine-glycine-aspartate; SαV, signal regulatory protein α variants; TeEV, targeting miR-222-engineered extracellular vesicle; TNB, targeted nanobubble.

##### 3.4.2.2 Targeting strategies of engineered EVs *in vivo*

The precise targeting of engineering EVs to the injured heart tissue was a prerequisite for the treatment of IHD. Therefore, the use of targeted strategies was particularly important. The pathological microenvironment of ischemic myocardium had unique molecular characteristics, including specific antigens exposed on the surface of damaged cardiomyocytes (such as MLC3), activated platelet deposition, and infiltration of inflammatory cells, etc. These characteristics provided a biological basis for the active targeting design of EVs. From the perspective of the modification methods of EVs, 10 studies used membrane fusion methods (such as platelet membrane, macrophage membrane, or neutrophil membrane) to enhance their targeting ability (23, 33, 35, 43, 50, 51, 53, 57, 60, 65). The core mechanism of this strategy lied in “using the membrane as a guide”. It not only endowed EVs with their inherent ability to organize and return to tissues, but also utilized immune regulatory molecules on the membranes of platelets, macrophages, or neutrophils (such as CD47, P-selectin) to achieve damage repair and inflammation elimination at the site of heart lesions, prolonged the half-life of circulating EVs and reduced the clearance by the mononuclear phagocytic system. 12 studies achieved their targeting purpose by surface modification. Among them, 9 studies used surface modification with targeting peptides, which mainly included T-peptide (APWHLSSQYSRT) (21), CMP(WLSEAGPVVTVRALRGTGSW) (37, 44), CHP/CTP/IMTP(CSTSMLKAC) (22, 28, 30, 40, 41, 49). The targeting mechanisms of these short peptides were diverse: the T-peptide achieved tissue anchoring by recognizing the upregulated fibronectin fragments in the extracellular matrix of cardiomyocytes; CMP mimicked part of the sequence of troponin T and could specifically bind to the structural components of damaged cardiac muscle sarcomeres; while the CSTSMLKAC type peptide segments tended to target the stress-related membrane proteins that were overexpressed in ischemic areas. These targeting peptides could enhance the targeting ability of EVs to the heart tissue. 2 studies employed anti-MLC3 antibody (21, 50), as MLC3 served as a surface marker specific to damaged myocardium, enabling targeted delivery. These targeting peptides and anti-MLC3 antibodies enabled EVs to specifically target the lesion sites of myocardial ischemia, thereby increasing the distribution of EVs in the myocardial tissue. Additionally, 1 study used anti-CD81 antibody and cRGD modification (31) to promote the adhesion of EVs to cells or cell matrices, enhancing the stability and retention rate of EVs in the heart.

Regarding administration routes, 28 studies employed *in situ* delivery to achieve targeted drug delivery to ischemic lesion sites in animal hearts. Compared with tail vein injection, *in situ* delivery (including cardiac injection, cardiac implantation, and pericardial injection) enabled engineered EVs to reach the lesion sites of the heart more quickly and precisely, which was the most direct and effective targeting strategy. Finally, there were still 5 studies that did not use targeting strategies (29, 31, 32, 60, 68). The specific details were shown in Table 4.

##### 3.4.2.3 Tracing experiments of engineered EVs *in vivo*

The *in vivo* tracing experiments of engineered EVs truly reflected their biological distribution, retention time and metabolic status in the animal bodies, which was of great significance for studying the therapeutic effects of engineered EVs. As shown in Table 4, IVIS imaging system, *in vivo* optical imaging instrument, infrared imaging system, immunofluorescence and flow cytometry analysis were the main methods for studying the *in vivo* tracing of engineered EVs. 44 studies used lipophilic fluorescent dyes (such as DiR, DiL, DID, DIO, Cy5.5, PKH26 or NIR815) or radioactive dyes to label extracellular vesicles, and then performed *in vivo* tracking of EVs using fluorescence imaging systems. 38 studies showed that compared with un-engineered EVs, the engineered EVs could preferentially reach myocardial tissue or cells and detect significant fluorescence signals; EVs effectively accumulated or continuously released in ischemic or infarcted areas, and had a longer retention time than other organs. 20 studies conducted *in vivo* multi-organ tracing and imaging. The results showed that the engineered EVs labeled with markers could be found in organs such as liver, spleen, heart, lung, kidney, brain and bone marrow, but most were distributed in the heart, liver and spleen. In addition, 12 studies did not conduct *in vivo* tracing experiments, so it was impossible to analyze the situation of engineered EVs reaching the lesion sites (32, 36, 39, 44, 45, 47, 48, 54, 58, 66, 67).

#### 3.4.3 *In vivo* therapeutic effects of engineered EVs in animals with IHD

Engineering EVs, as a new type of drug delivery carrier, with their carrying of signaling molecules, excellent biocompatibility and the advantage of precise delivery, had shown broad application prospects in the treatment of IHD. Based on a systematic analysis of 50 studies, we established that engineering EVs had multiple therapeutic effects on animals with IHD. For example: improving cardiac function (including multiple cardiac function indicators such as LVEF, LVFS, etc.), alleviating myocardial fibrosis (mainly reflected by protein molecules related to myocardial fibrosis, such as Col1a1, α-SMA, MMP9, etc.), inhibiting cardiac inflammation (including anti-inflammatory and anti-inflammatory related signaling molecules and cell quantities, such as IL-10, CTLA-4, iNOS, TNF-α, etc.), promoting angiogenesis (including genes or proteins related to angiogenesis, such as VEGFA, CD31, PCNA, etc.), reducing apoptosis (including cell apoptosis-related signaling molecules and cell quantities, such as GDF-15, miR-214–3p, cTnI⁺ Tunel⁺, etc.), optimizing mitochondrial metabolism (including mitochondrial morphology and function, antioxidant enzymes and oxidative stress markers, such as SOD, ROS, malondialdehyde, etc.). This indicated that engineered EVs exerted multiple cardioprotective effects and demonstrated therapeutic potential *in vivo*. More detailed indicators were presented in the following 6 subsections. Specific details and abbreviations could be found in Table 5.

**Table 5:** The therapeutic effects of engineered EVs in animal models of IHD.

**Author, year, country**	**Animal strain/disease types**	**Groups**	**Results of cardiac function indicators**	**Results of fibrosis indicators**	**Results of inflammatory indicators**	**Results of angiogenesis indicators**	**Results of apoptosis indicators**	**Results of mitochondrial and metabolism indicators**
Mun, 2026, Korea (21)	Mice/MI	Sham, MI, MI + T-MNV, MI + mNC@T-MNV, MI + m10@T-MNV	LVEF, LVFS↑; LVEDV, LVESV↓.	Infarction area, fibrosis area, collagen deposition, collagen I, α-SMA↓.	Heart: iNOS, TNF-α↓; TGF-β, Arg1, CD206↑.		Heart: Tunel + cells↓.	
Luo, 2026, China (22)	Mice/MI	Sham, MI, MI + EXO, MI + EXO-(IC-L), MI + EXO-(IC-LN), MI + EXO-(IC-LNV)	LVEF, LVFS↑; LVIDs↓.	Infarction area, fibrosis area, collagen I and Acta2 mRNA, collagen I and α-SMA↓.	Heart: Arg1, Chil3, Mrc1, IL-10↑.	Heart: the number and density of blood vessels and arterioles, the migration rate of HUVECs↑.		Heart: the destruction of the mitochondrial spine ↓, the density of the mitochondrial cristae and the activity of mitochondrial complex I ↑.
Liu, 2026, China (23)	Rat/MI	Sham, MI, MI + Hy-Nvs, MI + Hy-GNvs@chol	LVEF, LVFS, LVAWd, LVAWs↑; LVIDd, LVIDs, EDV, ESV↓.	Infarction area, fibrosis area↓.	Heart:CD68 + CD86+ M1 macrophages↓; CD68 + CD206+ M2 macrophages↑.	Heart: the number of blood vessels, α-SMA, CD31↑.	Heart: Tunel + cells↓.	
Zhou, 2025, China (24)	Mice/MI	Sham, MI, MI + GqDNVs-Gel	LVEF, LVFS↑, LVEDV, LVESV↓.	Infarction area, fibrosis area, TGFβ-2, α-SMA, col3a1, col1a1 mRNA↓; IGF1, VEGFA↑.		Heart:CD31↑.	Heart: Tunel + cells; Bax, Caspase-7, Caspase-3 mRNA; Bax, cleaved Caspase-7, cleaved Caspase-3↓.	Heart: metabolomics involves glycerophospholipid metabolism, linoleic acid metabolism and α-linolenic acid metabolism pathways.
Zhao, 2025, China (25)	Rat/ (MI/R)	Sham, MI/R, MI/R + Rg1-ACDVs	LVEF, LVFS↑.	Infarction area, fibrosis area↓.			Heart: Tunel + cells↓.	Heart: ROS↓. Serum: MDA↓; SOD、CAT、GSH↑.
Yang, 2025, China (26)	Rat/ (MI/R)	Sham, MI/R, MI/R + Exo-WT, MI/R + Exo-I-S	LVEF, LVFS↑.	Infarction area↓.				Heart: ROS↓; SOD2, GLUT4↑.
Wei, 2025, Singapore (27)	Mice/ (MI/R)	Sham, MI/R, MI/R + NV	LVEF↑; LVEDV, LVESV↓.	Infarction area↓.				
Wang, 2025, China (28)	Mice/ (MI/R)	Sham, MI/R, MI/R + GEL, MI/R + TeEV, MI/R + GEL-TeEV	LVEF, LVFS↑.	Infarction area, fibrosis area, col1a1 and col3a1 mRNA, α-SMA↓.	Heart: TNF-α, IL-1β, IL-6↓.		Heart: Tunel + cells, the ratio of Bax/Bcl-2 and cleaved Caspase-3/Caspase-3↓.	
Wang, 2025, China (29)	Mice/MI	MI + PBS, MI + sEV, MI + C@sEV, MI + CM@sEV	LVEF, LVFS↑.	Infarction area, fibrosis area, collagen area↓.				
Wang, 2025, China (30)	Rat/ (MI/R)	Sham, MI/R, MI/R + PBS, MI/R + Control-EXO, MI/R + Blank-EXO, MI/R + IMTP-EXO	LVEF, LVFS↑; LVEDD, LVESD↓.	Infarction area↓.	Heart: MCP-1, IL-1β, TNF-α↓; CD206↑.	Heart: the number of capillaries, α-SMA↑.		
Wang, 2025, China (31)	Rat/MI	Sham, MI, MI + TNB, MI + EXO, MI + TNB + EXO, MI + EXO SDF-1α, MI + EXO SDF-1α+TNB	LVEF, LVFS↑.	Fibrosis area↓.	Heart: neutrophil, CD68 + CD86+ M1 macrophages, IL-6↓; CD68 + CD206+ M2 macrophages, IL-10↑. Serum:IL-6↓; IL-10↑.	Heart: the density of arterioles, α-SMA↑.	Heart: Tunel + cells↓.	
Liu, 2025, China (32)	Mice/ (MI/R)	Sham, MI/R, MI/R + NV, MI/R + NV-inhibitor, MI/R + NV-mimics	LVEF, LVFS↑.	Fibrosis area↓.		Heart: the number of new small blood vessels and arterioles↑.	Heart: Tunel + cells↓.	Heart: breakage of the mitochondrial spine↓. Serum: MDA↓; SOD, GSH↑.
Liu, 2025, China (33)	Mice/MI	Sham, MI, MI + P-NV, MI + P-181-NV	LVEF, LVFS, CO, SV↑; LV Vol d, LV Vol d↓.	Infarction area, fibrosis area↓.	Heart: CCR2 + pro-inflammatory macrophages↓; CX3CR1 + and CD206 + anti-inflammatory macrophages↑.	Heart:CD31, α-SMA↑.		
Li, 2025, China (34)	Mice/MI	MI, MI + dECM/GP, MI + dECM/GP@EVS, MI + dECM/GP@EVS@GPNMB, MI + EVS@GPNMB	LVEF, LVFS↑; LVEDV, LVESV, LVEDD, LVESD↓.	Infarction area, fibrosis area↓.	Heart: GPNMB + macrophages↑.		Heart: cleaved Caspase3↓; Bcl-2↑.	
Chen, 2025, China (35)	Mice/ (MI/R)	MI/R, MI/R + PBS, MI/R + NP, MI/R + Neu-NP	LVEF, LVFS↑; LVEDV, LVESV↓.		Heart: MPO + cells, TNF-α, IL-6↓.	Heart:CD31 + cells↑.		
Wang, 2024, China (36)	Mice/MI	Sham, MI, MI + Exo, MI + Exo-miR-223	LVEF, LVFS↑; LVIDd, LVIDs↓.	Infarction area↓.	Heart: TNF-α, IL-1β, IL-18, IL-6, NLRP3, cleaved Caspase-1↓.			
Mentkowski, 2024, America (37)	Mice/MI	MI, MI + PBS, MI + EVs-IM, MI + EVs-IV, MI + (CMP-EVs)-IM, MI + (CMP-EVs)-IV	LVEF↑.	Infarction area, fibrosis area↓.			Heart: cleaved Caspase3, the Percentage of apoptotic cardiomyocytes↓.	
Xue, 2024, China (38)	Mice/ (MI/R)	Sham, MI/R, MI/R + Mito Q, MI/R + MMNv, MI/R + Mito Q@MMNv	LVEF, LVFS↑.	Infarction area, fibrosis area, MMP9↓.	Heart:CD86+ M1 macrophages↓; CD163+ M2 macrophages↑.	Heart: CD31↑.	Heart: Tunel + cells.	
Zou, 2024, China (39)	SD rat/MI	Sham, MI + PBS, MI + NC-EV, MI + GDF-15-EV	LVEF, LVFS↑.	Infarction area, fibrosis area, collagen area↓.	Serum:IL-6, TNF-α, IL-10, NLRP3↓.	Heart: the density of capillaries↑.	Heart: Tunel + cells, cleaved Caspase-3↓; Bcl-2↑.	
Zhang, 2024, China (40)	Mice/MI	Sham, MI + PBS, MI + EV, MI + PLGF, MI + EV-PLGF, MI + EV-CHP, MI + EV-P-C	LVEF↑; LVIDd↓.	Infarction area, fibrosis area↓.	Heart: TNF-α, IL-6↓.	Heart: the density of capillaries and arterioles↑.		
Yin, 2024, China (41)	Mice/ (MI/R)	Sham, MI/R + siNC-EV, MI/R + siWTAP-EV, MI/R + siNC-EV-CHP, MI/R + siWTAP-EV-CHP	LVEF, +dp/dtmax, -dp/dtmin↑.	Infarction area↓.			Heart: Tunel + cells↓.	Heart: TXNIP mRNA m6A, TXNIP, MDA↓; the activity of SOD↑.
Tan, 2024, China (42)	Mice/MI	Sham, MI, MI + HA, MI + EV, MI + HA-EV	LVEF, LVFS↑; LVDs, LVDd↓.	Infarction area, fibrosis area↓.	Heart:IL-6 + and TNF-α+cells↓; CD206+ M2 macrophages, the M2/M1 ratio↑.	Heart: the density of capillaries↑.	Heart: Caspase3 + cells↓.	
Lai, 2024, China (43)	Mice/ (MI/R)	Sham, MI/R + PBS, MI/R + PLT-NV, MI/R + EXOs, MI/R + SαV-NVs, MI/R + hNVs	LVEF, LVFS↑.	Scar size↓.	Heart:M1 macrophages, SHP-1, p-SHP-1, IL-1β, TNF-α↓. Serum: IL-1β, TNF-α↓.		Heart: Tunel + cells↓.	
Gu, 2024, China (44)	Mice/ (MI/R)	Sham, MI/R, MI/R + DSPE-PEG-CMP, MI/R + DSPE-PEG-CMP-EV, MI/R + miR302, MI/R + DSPE-PEG-CMP-miR302-EV	LVEF, LVFS, LV Mass Index, LVAWd, LVAWs↑; LV Vold, LV VOLs, LVIDd, LVIDs, LVPWd, LVPWs↓.	Infarction area↓.	Serum: cTnI, CKMB, TNF-α, IL-1β↓.		Heart: Tunel + cells, Bax↓; Bcl-2↑.	
Das, 2024, India (45)	Rat/MI	Sham, MI, MI + PUGA-dECM, MI + PUGA-dECM + EV	LVEF, LVFS↑; LVDs, LV VOLs↓.	Infarction area, fibrosis area, collagen content↓.	Heart: iNOS↓.	Heart:CD31, PCNA↑.		Heart:8OHdG↓.
Zhu, 2023, China (46)	Rat/MI	Sham, MI + PBS, MI + Ctrl-EV, MI + miR-214OE-EV	LVEF, LVFS↑.	Infarction area, fibrosis area, collagen I and collagen III↓.	Heart: iNOS + macrophages↓.	Heart: the density of capillaries and arterioles↑.	Heart: Tunel + cells, Caspase3 + cells↓; Cx43↑.	
Yu, 2023, China (47)	Mice/MI	MI + PBS, MI + EPC-EV, MI + microsphere + EPC-EV, MI + microsphere + CS-EPC-EV	LVEF, LVFS↑; LV VOLd, LV VOLs↓.	Infarction area, fibrosis area↓.		Heart: the number of capillaries and arterioles↑.	Heart: Tunel + cells↓.	
Lin, 2023, China (48)	Rat/MI	Sham, MI, MI + Se NPs, MI + EV, MI + hydrogel, MI + Se NPs-EV-hydrogel	LVEF, LVFS↑; LVIDd, LVIDs, EDV, ESV↓.	Infarction area, fibrosis area↓.	Heart: TNF-α, IL-1β, IL-6↓; IL-10, TGF-β↑.		Heart: Tunel + cells↓.	
Lee, 2023, Korea (49)	Rat/ (MI/R)	MI/R, MI/R + nNV, MI/R + ApoNV, MI/R + ApoNV-DC	LVEF, LVFS, +dp/dtmax, -dp/dtmin, ESPVR↑; LVIDd, LVIDs, EDPVR↓.	Fibrosis area↓.	Heart: NOS2+ M1 macrophages↓; CD206+ M2 macrophages↑.	Heart: the number of blood vessels↑.	Heart: the number of surviving cardiomyocytes↑.	
Huang, 2023, China (50)	Mice/ (MI/R)	Sham, MI/R, MI/R + CST, MI/R + CMEV, MI/R + CST@CMEV	LVEF, LVFS↑.	Infarction area, fibrosis area, collagen volume, collagen I, MMP9 and MMP2↓.	Heart:M1 macrophages, IL-1α, IL-1β, IL2, IL-6, IL-17α, TNF-α, IFN-γ, MCP-1, MIP-1α, MIP-1β, GCSF↓; M2 macrophages, IL-4, IL-10, IL-12, IL-13↑.		Heart: Tunel + cells↓.	Heart: ATP, morphology of mitochondria↑.
Chen, 2023, China (51)	Mice/MI	MI, MI + MmEVs, MI + Tβ4-MmEVs	LVEF, LVFS↑.	Infarction area, fibrosis area↓.		Heart: α-SMA, CD31, PH3↑.		
Bheri, 2023, America (52)	Rat/ (MI/R)	Sham, MI/R, MI/R + L-sEV, MI/R + H-sEV, MI/R + L-ELV, MI/R + H-ELV	LVEF, LVFS↑.	Infarction area↓.		Heart: the density and size of blood vessels↑.		
Zhong, 2023, China (53)	Mice/MI	MI, MI + iCM-EXs, MI + M-iCM-EXs	LVEF, LVFS↑; LVIDd, LVIDs↓.	Infarction area↓.				
Zhang, 2022, China (54)	Mice/MI	Sham, MI + PBS, MI + EVs, MI + Mel@EVs	LVEF, LVFS↑.			Heart:CD31 + cells↑.	Heart: Tunel + cells↓.	
Shiekh, 2022, India (55)	Rat/MI	Sham, MI, MI + PUAO, MI + PUAO-CPO-COL, MI + PUAO-CPO-COL + EV	LVEF, LVFS↑; LVIDd, LVIDs↓.	Fibrosis area, collagen deposition↓.		Heart:CD31 + cells↑.		Heart:8OHdG↓.
Monguió-Tortajada, 2022, Spain (56)	Pig/MI	MI, MI + buffer, MI + EV	RVEF↑.	Fibrosis area, collagen I, MMP2, MMP9, TIMP1↓.	Heart:CD163 + cells, CD73 + cells, TGF-β1, TNF-α↓; TGF-β3, IL-10, the ratio of IL-10/TNF-α ↑. Serum: TNF-α↓.			
Li, 2022, China (57)	Mice/ (MI/R)	MI/R + PBS, MI/R + EVs, MI/R + P-EVs	LVEF, LVFS↑; LVEDD, LVESD↓.	Infarction area, fibrosis area↓.	Heart:CD86 + macrophages, IL-1β, TNF-α↓; CD206 + macrophages, Arg-1, TGF-β↑. Serum:IL-6, TNF-α↓; TGF-β, IL-10↑.			
Hu, 2022, China (58)	Mice/MI	Sham, MI, MI + Ang-1 Gel, MI + MSCs EXO, MI + Islet-1 MSCs EXO, MI + Ang-1 Gel + Islet-1 MSCs EXO	LVEF, LVFS↑.	Fibrosis area↓.		Heart:CD31 + cells↑.		
Hao, 2022, China (59)	Mice/MI	Sham, MI, MI + PBS-ECM, MI + EV-ECM	LVEF, LVFS↑.	Infarction area↓.	Heart: the area of cardiac infarction and mediastinal lymph nodes, the ratio of Tregs cells/leukocytes↓; the ratio of MHC-II macrophages/macrophages, the ratio of Tregs cells/T cells↑.	Heart:CD31 + cells↑.		
Bao, 2022, China (60)	Rat/MI	MI, MI + eNAB^null^, MI + MSN^HAL^, MI + eNAB^HAL^	LVEF, LVFS↑.	Infarction area, fibrosis area↓.	Heart: iNOS + macrophages, neutrophils, iNOS↓; CD206 + macrophages, CD206, Arg1↑.	Heart:CD31 + cells↑.		
Yuan, 2022, China (61)	Mice/ (MI/R)	Sham, MI/R, MI/R + EV, MI/R + EV-NC, MI/R + EV-miRNA-204 mimic	LVEF, LVFS↑; LVEDD, LVESD↓.	Infarction area↓.	Heart: macrophages, CD11c, IL-1β, TNF-α↓; CD206, Arg-1, IL-10↑.			
Zhu, 2021, China (62)	Rat/MI	Sham, MI + PBS, MI + siMIF-EV, MI + EV, MI + MIF-EV	LVEF, LVFS↑.	Fibrosis area↓.		Heart: the density of capillaries and arterioles↑.	Heart: Tunel + cells↓.	
Wang, 2021, China (63)	Rat/MI	MI + PBS, MI + NC-EVs, MI + HIF-1α-EVs, MI + HIF-1α-EVs-RGD	LVEF, LVFS↑.	Infarction area, fibrosis area↓.	Heart: IL-6↓.	Heart: the density of capillaries and arterioles↑.	Heart: Tunel + cells↓.	
Monguió-Tortajada, 2021, Spain (64)	Pig/MI	MI + hydrogel, MI + EV + hydrogel		Fibrosis area↓.	Heart: monocytes and T cells↓.	Heart: the density of blood vessels↑.		
Li, 2021, China (65)	Mice/ (MI/R)	MI/R + PBS, MI/R + EV, MI/R + P-EV	LVEF, LVFS↑; LVEDD, LVESD↓.	Infarction area, fibrosis area↓.		Heart: the density of capillaries and arterioles, pro-angiogenic genes (Tgfbr1, Flt1, Cxcl12, Kdr) ↑.	Heart: Tunel + cells↓.	
Kwon, 2021, America (66)	Mice/MI	Sham, MI + PBS, MI + GFP-EVs, MI + MEF-EVs, MI+βARKct-EVs	LVEF↑; LVIDs↓.	Fibrosis area↓.	Heart: neutrophils, IL-6, MCP-1, CCL-4↓. Serum: cTnI, IL-6↓; IL-10↑.		Heart: Tunel + cells↓.	
Xuan, 2020, America (67)	Mice/MI	MI + PBS, MI + EV-Notch1 FF, MI + EV-Notch1 KO, MI + EV-Notch1 CD	LVEF, LVFS↑.	Fibrosis area↓.		Heart: the density of blood vessels↑.	Heart: Tunel + cells↓.	
Wang, 2020, America (68)	Mice/MI	MI + PBS, MI + eEV, MI + miR-101a eEV	LVEF, LVFS↑; LVIDs, LVESV↓.	Infarction area, collagen content↓.	Heart: TGF-β1↓; the ratio of CD206/iNOS cells↑.			
Huang, 2020, China (69)	Rat/MI	Sham, MI, MI + EV, MI + ATV-EV	LVEF, LVFS↑; LVEDV, LVESV↓.	Infarction area, fibrosis area, col1a1 and col3a1↓.	Heart:IL-6, TNF-α↓.	Heart: the density of capillaries and arterioles↑.	Heart: Tunel + cells, cleaved Caspase-3 + cells↓.	
Song, 2019, China (70)	Mice/MI	Sham, MI + PBS, MI + EVs, MI + miR21-EVs	LVEF, LVFS↑; LVEDD, LVESD, LVEDV, LVESV↓.	Infarction area, fibrosis area↓.		Heart: the density of capillaries and arterioles↑.	Heart: PDCD4 + cells, (cTnI + Tunel+) cells, (CD31 + Tunel+) cells↓.	

ACDVs, artificial cell derived vesicles; Acta2, Actin Alpha 2, smooth muscle; Ang-1, angiogenin-1; ApoNV, apoptotic body-mimetic nanovesicle derived from apoptotic fibroblast; ApoNV-DC, ApoNVs conjugated with dextran and ischemic cardiac homing peptide (CHP); Arg1, Arginase 1; α-SMA, α-smooth muscle actin; ATP, adenosine triphosphate; ATV, atorvastatin; βARKct, β-adrenergic receptor kinase 2 c-terminal tail; Bax, bcl-2 associated X protein; Bcl-2, B-cell lymphoma 2; CAT, catalase; CCL-4, chemokine (C-C motif) ligand 4; CCR2, C-C motif chemokine receptor 2; Chil3, chitinase-like 3; chol, cholesterol; CHP, ischemic heart homing peptide, CSTSMLKAC; CKMB, creatine kinase isoenzymes; CMEV, cellular membrane-engineered nanovesicle; CMP, cardiomyocytes specific peptide, WLSEAGPVVTVRALRGTGSW; CO, cardiac output; col1a1, collagen, type I; col3a1, collagen, type III; CST, catestatin; cTnI, cardiac troponin I; Cxcl12, C-X-C motif chemokine ligand 12; CX3CR1, C-X3-C motif chemokine receptor 1; Cx43, connexin 43; dECM/GP, dextran-peptide hydrogel; +dp/dtmax, positive first derivative of pressure max; -dp/dtmin, negative first derivative of pressure min; ECM, extracellular matrix; EDPVR, end-diastolic pressure-volume relationship; EDV, end-diastolic volume; eNABs, engineered neutrophil apoptotic bodies; EPC, endothelial progenitor cell; ESPVR, end-systolic pressure-volume relationship; ESV, end-systolic volume; EVs, extracellular vesicles; Flt1, fms-like tyrosine kinase 1; GCSF, granulocyte colony-stimulating factor; GDF-15, growth differentiation factor-15; GFP, green fluorescent protein; GLUT4, glucose transporter type 4; GPNMB, glycoprotein non-metastatic melanoma protein B; GqDNVs, loaded Gouqi-derived nanovesicles; GSH, glutathione; HA, hyaluronic acid; HAL, hexyl 5-aminolevulinate hydrochloride; HIF-1α, hypoxia-inducible factor-1α; HUVECs, human umbilical vein endothelial cells; IFN-γ, interferon gamma; IGF1, insulin-like growth factor 1; IL-6, interleukin-6; IL-10, interleukin-10; iNOS, nducible nitric oxide synthase; Kdr, kinase insert domain rreceptor; LVAWd, left ventricular anterior wall thickness (diastole); LVAWs, left ventricular anterior wall thickness (systole); LVDd, left ventricular dimension (diastole); LVDs, left ventricular dimension (systole); LVEDD, left ventricular end-diastolic diameter; LVEDV, left ventricular end-diastolic volume; LVEF, left ventricular ejection fraction; LVESD, left ventricular end-systolic diameter; LVESV, left ventricular end-systolic volume; LVFS, left ventricular fractional shortening; LVIDd, left ventricular internal dimension (diastole); LVIDs, left ventricular internal dimension (systole); LVPWd, left ventricular posterior wall thickness (diastole); LVPWs, left ventricular posterior wall thickness (systole); LV Vol d, left ventricular volume (diastole); LV Vol s, left ventricular volume (systole); MCP-1, monocyte chemoattractant protein-1; MDA, malondialdehyde; MEF, the primary mouse embryonic fibroblast; MHC-II, major histocompatibility complex class II; MI, myocardial infarction; MIP-1α, macrophage inflammatory protein-1 alpha; MIP-1β, macrophage inflammatory protein-1 beta; miR, microRNA; MI/R, myocardial ischemia/reperfusion; Mito Q, a well-established mitochondrial-targeted antioxidant; MmEVs, cell membrane-modified extracellular vesicles; MMP2, matrix metallopeptidase 2; MMP9, matrix metallopeptidase 9; MPO, myeloperoxidase; Mrc1, mannose receptor c-type 1; MSCs, mesenchymal stem cells; Neu-NP, neutrophil membrane-coated poly lactic-co-glycolic acid nanoparticle; NLRP3, NLR family pyrin domain containing 3; NOS2, nitric oxide synthase 2; Notch1, Notch receptor 1; Notch1 CD, Notch1 overexpressing; Notch1 FF, Notch1 flox; Notch1 KO Notch1 knockout; NV, nanovesicle; 8OHdG, 8-hydroxy-2′-deoxyguanosine; PCNA, proliferating cell nuclear antigen; PDCD4, programmed cell death 4; PH3, phosphohistidine; PEG, polyethylene glycol; PLGF, placental growth factors; PLT, platelet; p-SHP-1, phosphorylated SH2 domain containing protein tyrosine phosphatase-1; PUGA, polyurethane modified with antioxidant gallic acid in its backbone; Rg1, ginsenoside Rg1; RGD, arginine-glycine-aspartate; ROS, reactive oxygen species; RVEF, right ventricular ejection fraction; SαV, signal regulatory protein α variants; SDF-1α, stromal cell-derived factor-1 alpha; Se NPs, selenium nanoparticles; SHP-1, SH2 domain containing protein tyrosine phosphatase-1; siWTAP, small interfering RNA targeting WTAP; SOD, superoxide dismutase; SV, stroke volume; Tβ4, thymosin β4; TeEV, targeting miR-222-engineered extracellular vesicle; TGF-β, transforming growth factor beta; Tgfbr1, transforming growth factor beta receptor 1; TIMP1, TIMP metallopeptidase inhibitor 1; TNB, targeted nanobubble; TNF-α, tumor necrosis factor-α; TXNIP, thioredoxin interacting protein; VEGFA, vascular endothelial growth factor A.

##### 3.4.3.1 Improving cardiac function

In this systematic review, all studies employed ligation of the left anterior descending (LAD) to establish animal models of IHD: 33 constructed myocardial infarction models and 17 established ischemia-reperfusion injury models. Except for one study (64), the remaining 49 studies all detected cardiac function through echocardiography. After intervention with engineering EVs, the left ventricular systolic and diastolic function, cardiac chamber mechanical indicators of the animals were significantly improved, manifested as significant improvements in multiple cardiac function indicators such as ejection fraction, ventricular wall thickness, cardiac output and myocardial contraction and relaxation rates (LVEF, LVFS, LVAWd, LVAWs, CO, SV, +dp/dtmax, -dp/dtmin, LV Mass Index, ESPVR, RVEF), while indicators related to left ventricular volume, chamber diameter and myocardial stiffness were significantly reduced (LVEDV, LVESV, LVIDd, LVIDs, LVEDD, LVESD, LV Vol d, LV Vol d, LVPWd, LVPWs, EDPVR). The overall cardiac structure, and systolic and diastolic functions were significantly restored.

##### 3.4.3.2 Anti-myocardial fibrosis

Among the 50 studies, the signaling molecules loaded or knocked out inside EVs and modified on their membrane surfaces mainly included cytokines, transcription factors, growth factors, enzymes, peptides, miRNAs, and drugs. Among them, miR-214-3p (46), Catestatin (50), miR-101a-3p (68), and miR-21 (70) were closely related to the pathological process of cardiac fibrosis. After the treatment with engineered EVs, the myocardial infarction area, fibrosis area, and collagen deposition in the model group animals were significantly reduced. At the same time, engineered EVs inhibited the expression of pro-fibrotic genes or proteins (Col1a1, Col3a1, α-SMA) by the signaling molecules they carried, downregulated the content of matrix remodeling-related molecules (MMP2, MMP9, TIMP1), reduced the activity of the TGFβ-2 signaling pathway, and upregulated the expression of pro-repair factors (IGF1, VEGFA). Finally, it achieved the anti-fibrotic effect of reducing collagen deposition, improving myocardial fibrosis, and promoting cardiac remodeling after injury, providing a therapeutic idea for the reversal of myocardial fibrosis after ischemic heart disease.

##### 3.4.3.3 Inhibiting inflammatory response

Inflammatory response was the core link affecting the pathological progression of IHD, and the signaling molecules with anti-inflammatory effects included IL-10 (31, 39, 48, 50, 56, 58, 61, 66), CTLA-4 (29), SDF-1α (31), miR-181a-5p (33), miR-223-3p (36), GDF-15 (39), SIRPα (43), miR-214-3p (46), Catestatin (50), miR-204 (61), and MIF (62). In addition, the modification of the fusion of macrophage and neutrophil membranes was also an important means to alleviate inflammatory response. They mainly exerted anti-inflammatory effects by regulating inflammatory cytokines and chemokines, promoting the transformation of macrophages to an anti-inflammatory phenotype. Anti-inflammatory effect specifically included: reducing the expression of pro-inflammatory factors (iNOS, TNF-α, IFN-γ, IL-1β, IL2, IL-6, IL-17A, IL-18, MCP-1, NLRP3, MIP-1α/β, CCL-4, GCSF) and the number of pro-inflammatory cells (CD68^+^CD86^+^, iNOS^+^, NOS2^+^, CCR2^+^ cells), while increasing the levels of anti-inflammatory factors (TGF-β, Arg1, IL-4, IL-10, IL-12, IL-13) and the number of anti-inflammatory cells (CD68^+^CD206^+^, CD163^+^, CX3CR1^+^ cells), thereby improving the immune response and inflammatory process.

##### 3.4.3.4 Promoting angiogenesis

Angiogenesis was a key link in the repair of IHD. Engineered EVs effectively activated angiogenesis in ischemic myocardium by loading various angiogenic molecules and modifying their membrane surfaces. Specifically, VEGFA (22, 24), miR-222 (28, 32), SDF-1α (31), PLGF (40), miR-214-3p (46), Tβ4 (51), miR-126 (52), HIF-1α (63), Notch1 (67), and angiogenin-1 (58) upregulated the expression of angiogenesis-related genes or proteins, enhanced endothelial cell function and increased the number of smooth muscle cells, thereby promoting angiogenesis. Additionally, platelet membrane fusion also enhanced vascular repair and regeneration, particularly in ischemia-reperfusion injury models. Following engineered EV intervention, cardiac capillary and arteriole density significantly increased, accompanied by upregulated expression of angiogenic markers (α-SMA, CD31, PCNA, PH3, Tgfbr1, Flt1, Cxcl12, and Kdr) and elevated CD31+ cell populations. These results demonstrated that engineered EVs effectively modulated angiogenic networks through multiple pathways, reconstructed blood supply to ischemic myocardium and represented a promising strategy for cardiac vascular regeneration in IHD.

##### 3.4.3.5 Reduction of apoptosis

MiR-222 (28, 32), GDF-15 (39), miR-214-3p (46), melatonin (54), βARKct peptide (66), and miR-21 (70) effectively attenuated apoptosis in cardiac and vascular cells. Following engineered EV intervention, pro-apoptotic proteins and genes (Bax, Caspase-3, Caspase-7, and their cleaved forms) were significantly downregulated, whereas anti-apoptotic Bcl-2 and Cx43 expression was upregulated. Concordantly, Tunel⁺, Caspase-3⁺, cleaved Caspase-3⁺, PDCD4⁺, cTnI⁺Tunel⁺, and CD31⁺Tunel⁺ cell populations markedly decreased. These results demonstrated that engineered EVs inhibit aberrant cardiomyocyte and endothelial apoptosis by modulating apoptosis-related molecular networks, thereby mitigating ischemic injury and preserving myocardial structural integrity.

##### 3.4.3.6 Improvement of mitochondrial metabolism

Mitochondrial dysfunction and oxidative stress represented critical determinants of the cellular microenvironment in IHD. Enhancing mitochondrial bioenergetics and bolstering antioxidant defenses constituted key therapeutic strategies for cardio-protection and attenuation of adverse cardiac remodeling. Notably, signaling molecules including Ndufs1 (22), SIRT3 (26), insulin (26), miR-222-3p (32), GPNMB (34), MitoQ (38), WTAP (41), and Catestatin (50) had demonstrated significant mitochondrial protective and antioxidant capacities. Compared with model group, engineered EVs treatment markedly ameliorated mitochondrial morphology and cristae density, restored complex I activity, and elevated ATP levels in cardiac tissue. Concurrently, EVs intervention upregulated the protein expression of GLUT4 and SOD2 protein expression, enhanced serum antioxidant enzyme activities (SOD, CAT), and increased GSH content. Furthermore, mitochondrial fragmentation was reduced, accompanied by significant attenuation of oxidative stress markers—including ROS, malondialdehyde, TXNIP, and 8-OHdG—in the myocardium. By encapsulating the above key signaling molecules, engineered EVs effectively ameliorated mitochondrial structure and function, bolstered cellular antioxidant capacity, and attenuated oxidative stress. These effects collectively maintained myocardial energy metabolic homeostasis, creating a favorable microenvironment conducive to metabolic reprogramming in IHD.

#### 3.4.4 Joint analysis of the diversity of therapeutic effects and the modification methods of EVs

The breadth of the therapeutic spectrum determined the comprehensiveness of engineered EVs intervention in IHD pathophysiology, ultimately influencing disease progression and clinical outcomes. Our systematic analysis of 50 studies identified 6 core therapeutic modalities: enhanced cardiac function, attenuated myocardial fibrosis, reduced inflammation, promoted angiogenesis, inhibited apoptosis, and regulated mitochondrial metabolism. Given that individual studies reported 2–5 concurrent therapeutic effects, we stratified them into dual-, triple-, quadruple-, and quintuple-modal therapy categories. Subsequently, we conducted a joint analysis of the 4 multi-modal therapies and the EVs modification methods to elucidate the intrinsic relationship and matching rules between the two, providing scientific basis for optimizing EVs design and maximizing therapeutic efficacy.

##### 3.4.4.1 The number of articles on the 4 multi-modal therapies

**Figure 2:**
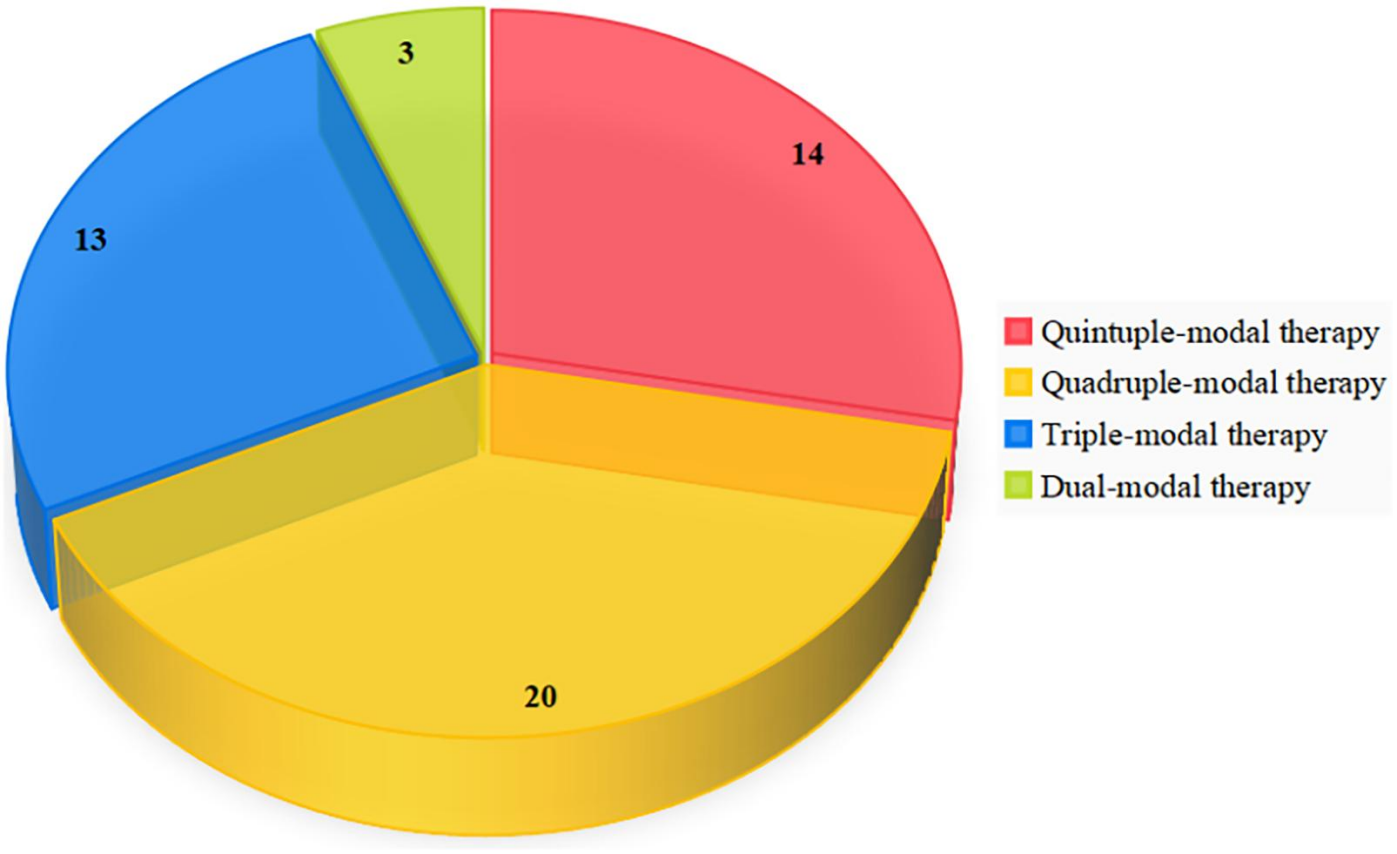
The number of articles on the 4 multi-modal therapies.

Figure 2 illustrated the distribution of publications across the 4 multi-modal therapeutic strategies. Quadruple-modal therapy represented the largest proportion of studies (20 articles, 40%), followed by quintuple-modal (14 articles, 28%) and triple-modal (13 articles, 26%). In contrast, dual-modal therapy accounted for the smallest share (3 articles, 6%). This distribution pattern suggested that collaborative interventions of multiple pathological links had emerged as the mainstream research direction for engineered EVs in ischemic heart disease treatment. Simple strategies targeting single or dual pathways were gradually being superseded, reflecting a growing emphasis on multi-modal regulation of the complex pathological processes underlying IHD to achieve more comprehensive cardioprotective effects.

##### 3.4.4.2 The specific composition of 4 multi-modal therapies

**Figure 3:**
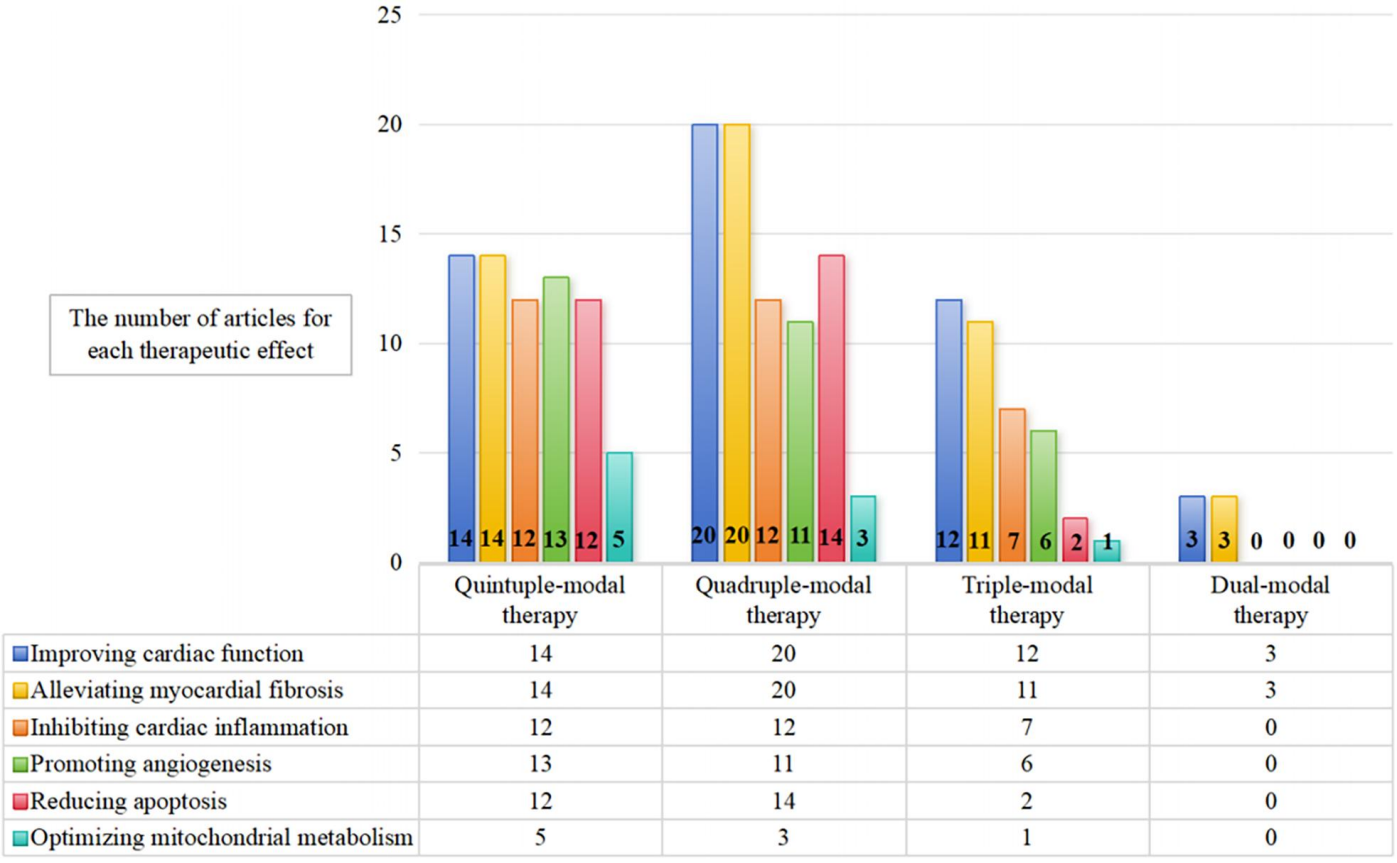
The specific composition of 4 multi-modal therapies.

To further elucidate the specific compositions of 4 multi-modal therapies, we systematically analyzed the distribution characteristics of 6 core therapeutic effects-improving cardiac function, alleviating myocardial fibrosis, inhibiting cardiac inflammation, promoting angiogenesis, reducing apoptosis, and optimizing mitochondrial metabolism-across the 4 multi-modal therapies, with corresponding article counts presented in Figure 3.

With the exception of dual-modal therapy, the remaining multi-modal therapies encompassed all 6 core therapeutic effects. First, cardiac function improvement was universally represented across all multi-modal therapies, as cardiac function served as the primary endpoint for evaluating treatment efficacy in IHD and constituted the critical criterion for assessing therapeutic success. Second, alleviation of myocardial fibrosis, inhibition of cardiac inflammation, promotion of angiogenesis, and reduction of apoptosis represented classical cardioprotective mechanisms. These four therapeutic effects were documented in 55%–100% of articles on quadruple- and quintuple-modal therapies. In triple-modal therapy, the proportions for anti-inflammatory, pro-angiogenic, and anti-apoptotic effects were 54%, 46%, and 15%, respectively. Finally, mitochondrial metabolic optimization showed the lowest representation, accounting for 36%, 15%, and 8% of articles on quintuple-, quadruple-, and triple-modal therapies, respectively. This pattern indicated that metabolic regulation represented an emerging therapeutic target with considerable potential for expanded application in higher-dimensional multi-modal therapies.

##### 3.4.4.3 Joint analysis of 4 multi-modal therapies and EVs modification methods

**Figure 4:**
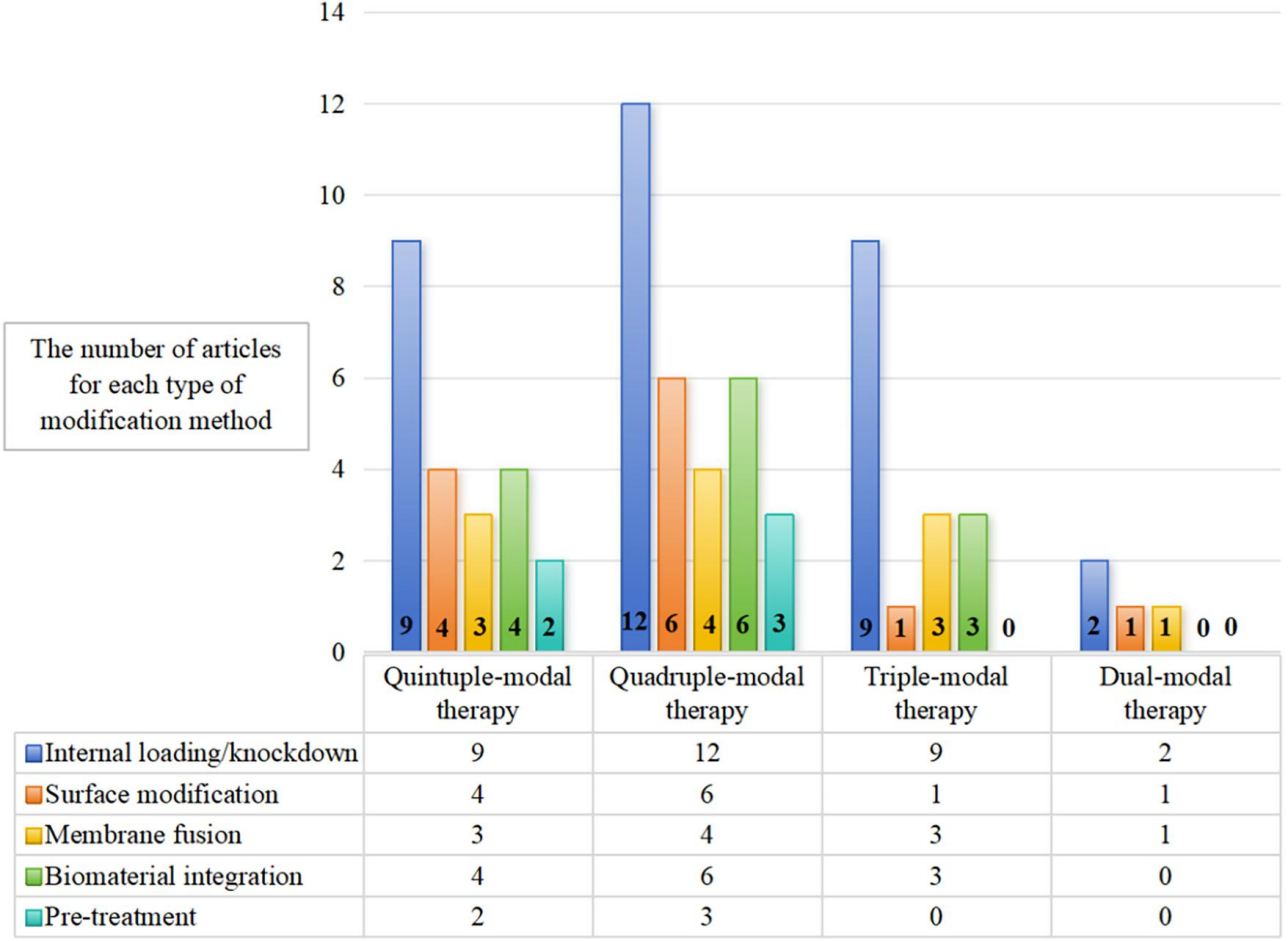
Joint analysis of 4 multi-modal therapies and EVs modification methods.

To further investigate how engineering modification methods influenced 4 multi-modal therapies, we performed a joint analysis of the distribution patterns of various modification techniques across each multi-modal therapy (Figure 4).

Internal cargo loading/knockdown emerged as the predominant modification strategy across all multi-modal therapies, representing 64% of quintuple-modal, 60% of quadruple-modal, 69% of triple-modal, and 67% of dual-modal, substantially exceeding the utilization rates of all other modification methods. This dominance likely reflected the high cargo capacity and versatile loading methodologies inherent to this technique. In quintuple- and quadruple-modal therapies, surface modification (29% and 30%, respectively) and integration with biological materials (29% and 30%, respectively) constituted the secondary tier of modification strategies, followed by membrane fusion (21% and 20%) and EVs preconditioning (14% and 15%). This hierarchical distribution suggested that high-dimensional multi-modal therapies employed internal cargo manipulation as the foundational strategy, while leveraging surface modification and membrane fusion to enhance targeting specificity, and biomaterial integration to achieve localized sustained release-thereby enabling multi-directional precision delivery of EVs. By contrast, triple-modal therapy relied primarily on internal loading/knockdown, with membrane fusion and biomaterial integration serving as ancillary strategies (23% each), whereas surface modification appeared less frequently (8%). This pattern indicated that triple-modal focused predominantly on intra-vesicular delivery of signaling molecules, optimizing delivery efficiency through biomaterial integration and membrane fusion, with minimal reliance on surface modification. Notably, dual-modal therapy exclusively utilized internal loading/knockdown (67%), surface modification (33%), and membrane fusion (33%), with no reported applications of biomaterial integration or EV preconditioning. This restricted repertoire further underscored that lower-dimensional multi-modal therapies depended primarily on straightforward internal cargo manipulation, lacking the sophisticated modification strategies characteristic of higher-dimensional approaches.

## 4 Discussion

EVs had emerged as promising drug delivery vehicles for IHD therapy. However, unmodified EVs exhibited inherent limitations, including suboptimal therapeutic efficacy, limited stability, and insufficient targeting specificity. Engineering modifications addressed these challenges by enhancing cardiac tissue- or cell-specific targeting, improving stability and bioactivity, and enabling the precise delivery of signaling molecules for targeted interventions. This review systematically analyzed 50 animal studies to examine the targeting strategies, modification methodologies, and multi-modal therapeutic effects of engineered EVs. These findings provided a mechanistic basis for optimizing modification strategies and designing more efficient combinatorial therapeutic regimens.

### 4.1 Targeting strategy

Efficient homing to and accumulation at injury sites represented critical manifestations of engineered EVs targeting capability, directly determining therapeutic efficacy. Although tail vein injection remained the most widely employed administration route, systemically delivered EVs were rapidly sequestered by the mononuclear phagocytic system, resulting in diminished retention at target lesions and compromised *in vivo* targeting efficiency. To overcome this limitation and ensure precise delivery to cardiac tissues or specific cell populations, targeted modification strategies were essential-including surface modification, membrane fusion, and localized myocardial injection (71, 72).

#### 4.1.1 Membrane surface modification

Surface modification of EVs entailed the conjugation of signaling molecules-such as peptides, antibodies, or functional groups-to EVs membrane via chemical, physical, or biological approaches. Targeting peptides had garnered substantial attention in recent years owing to their enhanced specificity for particular tissues or organs. For instance, Pham et al. decorated EV surfaces with EGFR targeting peptides, enabling efficient blood-brain barrier penetration and improved tumor-specific delivery of anti-neoplastic agents, thereby significantly suppressing tumor growth (73).

In cardiovascular research, peptide-directed targeting had similarly emerged as a prevalent strategy for EVs engineering. This review identified 3 distinct cardiac-targeting peptides: T-peptide (APWHLSSQYSRT), CMP (WLSEAGPVVTVRALRGTGSW), and the CHP/CTP/IMTP family (CSTSMLKAC). Through covalent conjugation to EVs membranes, these peptides conferred specific affinity for cardiac tissues, facilitating enhanced cellular internalization and rapid accumulation within ischemic myocardium. Furthermore, the robust expression stability of these peptides effectively prolonged myocardial retention of EVs, maximizing therapeutic efficacy while minimizing off-target effects on peripheral organs (74–79). Additionally, antibodies against myosin light chain 3 (MLC3) represented another class of surface modification agents that conferred cardiac-specific targeting capabilities to EVs. During myocardial infarction, sarcolemmal rupture exposed cytoskeletal MLC isoforms that were normally intracellular, thereby generating a lesion-specific antigenic signature (50, 80). Surface functionalization with either targeting peptides or MLC3-specific antibodies significantly enhanced the homing specificity of engineered EVs to ischemic cardiac tissue, prolonging their retention at injury sites. This approach maximized therapeutic efficacy while minimizing off-target biodistribution and associated systemic side effects, representing a critical engineering strategy for optimizing cardiac-targeted drug delivery.

#### 4.1.2 Membrane fusion

Membrane fusion represented an effective strategy for enhancing the targeting specificity of engineered EVs. Recently, membranes derived from somatic cells-particularly blood cells and immune cells-had been extensively employed to functionalize EVs, conferring biological properties such as organ-specific homing and immune evasion (81). Platelet membranes offered distinct advantages for cardiac targeting and inherently possessed homing capabilities mediated by P-selectin glycoprotein ligand-1 (PSGL-1), facilitating the recruitment of endothelial progenitor cells to vascular injury sites (82–84). Additionally, platelets actively modulated inflammatory processes through cytokine and chemokine regulation (85). Platelet membrane-coated EVs (PM-EVs) preferentially accumulated at damaged vasculature within ischemic myocardium, where they promoted growth factor and chemokine release, upregulated pro-angiogenic gene expression, attenuated inflammatory responses, and ultimately enhanced myocardial repair (43, 83, 86–89). Thus, PM-EVs not only had platelet-derived endothelial targeting but also retained pro-angiogenic and immunomodulatory potential (57, 90, 91).

Beyond platelet membranes, EVs modified with mononuclear macrophage or neutrophil membranes similarly exhibited robust homing capacity toward injured myocardium (92, 93). These modified EVs preferentially localized to inflammatory microenvironments under stress conditions, suppressed pro-inflammatory mediator release, modulated macrophage phenotypic polarization, and evaded the clearance of engineering EVs by the mononuclear phagocyte system (94).

Collectively, cell membrane fusion-based engineering enabled EVs precise homing and targeted enrichment within ischemic myocardium by harnessing the intrinsic biological properties of source cells. This approach effectively regulated the local inflammation and promoted angiogenesis, offering a promising strategy for enhancing targeted delivery efficiency in IHD therapy.

#### 4.1.3 Intra-cardiac delivery

Beyond surface modification and membrane fusion, localized myocardial delivery represented a critical strategy for enhancing the cardiac specificity of engineered EVs. The route of administration fundamentally determined EV biodistribution, cardiac retention, and systemic clearance. While tail vein injection remained widely utilized, biodistribution studies demonstrated that intramyocardial injection significantly enhanced cardiac retention compared with intravenous or intracoronary routes (95). Our systematic review identified 30 studies employing localized cardiac delivery approaches, encompassing direct intramyocardial injection, intrapericardial administration, and implantation of EVs-loaded bioengineered materials. Direct myocardial injection enabled rapid, precise accumulation at injury sites while minimizing systemic dispersion. As a minimally invasive alternative to cellular transplantation or surgical interventions, this approach reduced operative trauma and associated complications. Nevertheless, localized injection presented distinct challenges, including uncertainties regarding sustained therapeutic availability at the injury site and potential off-target uptake by non-cardiac cells. Consequently, route selection should be tailored to specific animal models and EVs engineering strategies. Besides *in situ* cardiac injection, EVs-encapsulated bioengineered materials-including hydrogels, scaffolds, and cardiac patches-were emerging as sophisticated localized delivery platforms. These biomaterials conferred enhanced biocompatibility, prolonged myocardial retention, sustained bioactivity through their tunable physicochemical properties, and offering controlled-release kinetics that might overcome the rapid clearance associated with bolus injections.

Hyaluronic acid-based hydrogels could recapitulate the biochemical components and modulus of pericardial fluid while preserving the bioactivity of encapsulated nanoscale EVs. This minimally invasive pericardial delivery approach significantly prolonged myocardial retention of EVs (42). Collagen, a major constituent of native cardiac extracellular matrix (ECM), was FDA-approved for diverse tissue engineering (96). Collagen-based hydrogels, decellularized ECM scaffolds, cardiac scaffolds and patches provided temporary mechanical support to ischemic myocardium, enhanced stem cell adhesion, and facilitated localized delivery of bioactive cargo (48, 55, 59). Furthermore, combined with RGD peptides (arginine-glycine-aspartic acid) promoted integrin-mediated cell-ECM adhesion, representing a promising strategy for ischemic heart disease therapy (63, 97).

In summary, the optimal delivery strategy depended on the specific clinical or experimental condition. For administration methods, tail vein injection remained predominant, necessitating complementary targeting strategies such as surface modification with cardiac-homing peptides or antibodies, membrane fusion with platelets or macrophages to enhance tissue specificity. Conversely, intramyocardial injection minimized systemic clearance but carried risks of off-target uptake. Alternatively, implantation of EVs-integrated biomaterials-including hydrogels, scaffolds, and patches with tunable physicochemical properties-offered a new platform for localized, sustained delivery to injured myocardium.

### 4.2 Multi-target therapeutic effects

Ischemic heart disease arose from inadequate coronary perfusion, precipitating myocardial ischemia and hypoxic injury. Its pathophysiology was characterized by intricate interconnections among multiple pathological processes-including adverse fibrotic remodeling, inflammatory cascade activation, vascular endothelial dysfunction, cardiomyocyte apoptosis, and mitochondrial energy metabolism disruption. Single-target interventions failed to confer comprehensive cardio-protection, exhibiting substantial therapeutic limitations. Consequently, multi-link, multi-pathway collaborative strategies had emerged as the central paradigm in this field. Engineered EVs, distinguished by their exceptional biocompatibility, minimal immunogenicity, and efficient cargo delivery capabilities, represented ideal carriers for multi-target therapeutic strategies. Systematic characterization of the distribution patterns, specific compositions, and intrinsic associations with engineering modifications underlying these multi-modal therapeutic effects would provide a mechanistic foundation for optimizing EV design and facilitating clinical translation.

#### 4.2.1 The multi-therapy of engineered EVs is closely related to the pathological process of IHD

Understanding the pathophysiological process of ischemic heart disease was essential for rational design of engineered EVs. Upon initial coronary hypoperfusion, cardiomyocytes underwent metabolic disruption and oxidative stress imbalance, triggering accelerated apoptosis and functional cell loss-representing the initial events of ischemic injury (98, 99). Notably, despite its fundamental role in disease initiation, metabolic regulation remained underutilized in current multi-modal therapies, presenting a significant opportunity for future therapeutic development. As the cardiac microenvironment deteriorated, necrotic and disintegrated cell activated innate immune responses, driving inflammatory cell infiltration and pro-inflammatory cytokine release. This excessive inflammatory cascade exacerbated cardiomyocyte damage and apoptosis (100–102). Concurrently, compensatory fibroblast activation and collagen deposition leaded to adverse myocardial fibrosis (103–105), while endothelial injury and insufficient angiogenic factor expression impaired neovascularization, perpetuating tissue hypoxia and cellular death (106–109). Consequently, therapeutic strategies targeting fibrosis modulation, inflammation resolution, angiogenesis promotion, and apoptosis inhibition constituted the four pillars of modifying IHD therapies. These pathways had been highly integrated into triple-modal, quadruple-modal, quintuple-modal therapies. The above pathological changes collectively increased myocardial stiffness and impaired ventricular wall motion, ultimately resulting in diastolic and systolic dysfunction. Cardiac functional parameters served as direct surrogates for structural damage and represented the primary efficacy endpoints across all studies included in this review (110, 111).

In conclusion, multi-modal therapies employing engineered EVs exhibited a hierarchical architecture: cardiac function served as the foundational endpoint, classical cardioprotective pathways constituted the structural framework, and metabolic modulation represented an emerging supplementary dimension. This hierarchical pattern reflected the evolving sophistication in our understanding of IHD pathophysiology, with broad recognition that coordinated multi-link, multi-pathway intervention was essential for achieving comprehensive myocardial protection. These insights provided references for designing more efficacious and precisely targeted combinatorial therapeutic regimens.

#### 4.2.2 Combined analysis of multi-drug therapy and modification methods

Multi-modal therapies had emerged as the predominant treatment paradigm for engineered EVs, with therapeutic efficacy critically dependent on rational engineering modifications. Distinct modification strategies-including internal cargo loading/knockdown, surface modification, and biomaterial integration-exhibited markedly different technical profiles that directly governed cargo capacity, targeting efficiency, and *in vivo* retention, thereby determining ultimate therapeutic outcomes. Analyzing multi-modal therapies or modification approaches in isolation failed to yield actionable guidance for EV engineering strategies. Therefore, integrative analysis of two dimensions could clear delineate of matching rules between specific modification methods and multi-modal therapies. Joint analysis of 4 multi-modal therapies and EVs modification methods revealed that the widespread adoption of internal cargo loading/knockdown reflected its intrinsic technical advantages. Researchers leveraged lentiviral, plasmid, or adenoviral transfection systems to efficiently load or deplete diverse signaling molecules-including miRNAs, proteins, and small-molecule drugs—within EVs, establishing this as the foundational technology for achieving multi-modal therapeutic effects. As therapeutic dimensionality increased, modification complexity escalated correspondingly. In higher-dimensional strategies (quadruple- and quintuple-modal therapy), surface modification (quadruple: 6 articles; quintuple: 4 articles) and biomaterial integration (quadruple: 6 articles; quintuple: 4 articles) are employed with substantially greater frequency, constituting a hierarchical architecture: internal cargo loading/knockdown of core signaling molecules, complemented by surface modification and membrane fusion for enhanced targeting specificity, and biomaterial-mediated local sustained release. This integrated approach addressed the multifaceted requirements of multi-stage coordination, precision delivery, and prolonged therapeutic action. Conversely, lower-dimensional strategies (triple- and dual-modal therapy) relied predominantly on internal loading/knockdown as the sole or primary modification, with minimal auxiliary engineering.

In summary, engineered EVs multi-modal therapies and modification methods exhibited a clear intrinsic interdependence. The selection of engineering strategies directly governed therapeutic diversity, with rational modification design serving as the technical cornerstone for coordinated multi- modal therapies. Future investigations should prioritize optimization of high-dimensional combinatorial regimens through integration of emerging pathways-particularly metabolic modulation-innovation in modification technologies, and enhanced translational potential, thereby establishing more efficacious and precise therapeutic paradigms for ischemic heart disease.

### 4.3 Limitations of preclinical studies and challenges in clinical translation

All the studies included in this systematic review were based on murine and pig models. The murine MI and MI/R models had significant advantages in terms of cost and efficiency for mechanism exploration, dose screening, and optimization of engineering strategies, providing a foundation for the molecular mechanism analysis and proof-of-concept of engineered EVs. The pig MI model was highly similar to humans in terms of anatomical structure (coronary artery distribution, degree of collateral circulation), cardiac physiology (heart rate, cardiac output, coagulation system), and pathological remodeling patterns, and was a key species recognized by the FDA/EMA for preclinical studies (112). Existing studies had confirmed that engineered EVs could reduce infarct area by 25%–45%, improve left ventricular ejection fraction, and promote angiogenesis in murine and pig MI models. Although there was significant heterogeneity in EVs sources, formulation forms, and result reporting, the murine and pig models still represented the current frontier level of cardiac EVs research, and more large animal studies were needed to fill the gap between human application. It was worth noting that as cell-free biological agents, engineered EVs had significant advantages over stem cell transplantation: their immunogenicity was extremely low, avoiding immune rejection and long-term immunosuppression; there was no risk of tumor formation or arrhythmia; and they could be freeze-dried for storage, possessing great potential for clinical translation and application. However, moving from murine and pig models to clinical applications still required the establishment of standardized cardiac targeting strategies, validated manufacturing platforms in accordance with Good Manufacturing Practice (GMP), batch-to-batch quality control, and the optimal therapeutic window (113). These were the main development and research directions for the clinical application transformation of engineered EVs in the future.

Gender and age profoundly affected the pathological physiological process of IHD and the biological characteristics of EVs. The lack of representation of female and elderly subjects in preclinical and clinical cardiovascular studies was a well-known systematic problem, limiting the general applicability of the therapeutic results of engineered EVs. The murine models included in this systematic review were also dominated by male animals, reflecting the current common situation in the field of preclinical cardiovascular regenerative medicine (114). Specifically, the remodeling of extracellular matrix in female myocardial cells, increased vascular stiffness and diastolic dysfunction presented unique patterns. Changes in estrogen levels directly affected myocardial calcium handling, mitochondrial energy metabolism, and oxidative stress responses. More importantly, there were significant gender differences in the concentration of circulating EVs, cell sources, and cargo composition (such as specific miRNAs and proteins), meaning that the engineered strategies and doses optimized based on male IHD models might not directly extrapolate to female patients (115). Additionally, as the most important risk factor for IHD, aging not only leaded to a decrease in circulating EVs concentration and functional changes (such as older source EVs being more likely to activate pro-inflammatory immune cells, while younger EVs had stronger pro-repair capabilities), but also shaped the aging heart microenvironment characterized by cellular senescence, chronic inflammation, and the decline of endogenous repair mechanisms (115). Therefore, in the current evidence system dominated by young male rodents, the efficacy and safety of engineered EVs for female and elderly patients with IHD still had significant uncertainties. Future research urgently needed to include female animals and models with ovariectomy (simulating post-menopausal estrogen deficiency) to clarify the influence of sex hormone status on EVs myocardial homing, cargo release, and treatment response; At the same time, the regulatory effects of EVs on aging markers (such as p16, p21, and SASP factors) and their repair efficacy should be verified in aged mice (such as those aged 18–24 months) and in models with co-morbidities related to aging (such as hypertension and diabetes). At the level of clinical trial design, it was necessary to ensure that the ratio of female and elderly subjects matched the epidemiological distribution of IHD in the real world, thereby enhancing the external validity and clinical translational value of the engineered EVs treatment strategy.

## 5 Limitations

In addition, this study still had some limitations. Firstly, not all the included studies examined the vesicle distribution in various organs, thus it was impossible to compare the vesicle retention rates among different organs. Secondly, due to the large number of literatures, this systematic review only included the application of engineered EVs in IHD, and did not include all the literatures on cardiovascular diseases (such as atherosclerosis, hypertension, etc.). Finally, because of the diversity of models and inconsistent intervention measures, we adopted a qualitative synthesis instead of a quantitative method, and thus could not draw more accurate conclusions from the meta-analysis.

## 6 Conclusions

Firstly, this systematic review provided a comprehensive overview of engineered EVs modification, extraction, and identification. We categorized current engineering modification methods into 5 distinct types: internal loading/knockdown, surface modification, membrane fusion, biomaterial integration, and pre-treatment. Secondly, our analysis of targeting strategies and tracing experiments in animal models of IHD identified 3 most effective targeting strategies: membrane fusion, surface modification with cardiac-targeting peptides or anti-MLC3 antibodies, and *in situ* delivery of the heart. These strategies significantly improved precise targeting to ischemic myocardium. Subsequently, we classified the therapeutic effects of engineered EVs into 6 categories: improving cardiac function, alleviating myocardial fibrosis, inhibiting cardiac inflammation, promoting angiogenesis, reducing apoptosis, and optimizing mitochondrial metabolism. Finally, joint analysis of the intrinsic relationship between 4 multi-modal therapies and modification methods revealed fundamental principles governing engineered EVs efficacy. ① Multi-modal therapies exhibited a pyramid-like architecture: improving cardiac function served as the foundation, ameliorating classical cardioprotective pathways constituted the primary pillars, and optimizing metabolic modulation represented supplementary. ② The corresponding EVs modification strategies formed a hybrid modification system: internal cargo loading/knockdown of core signaling molecules + surface modification and membrane fusion to enhance targeting specificity + combination with bioengineering materials for local sustained release. This engineering modification strategies satisfied the multifaceted requirements of multi-link coordination, precision delivery, and prolonged therapeutic action. The systematic review established a solid scientific foundation for optimizing engineered EVs therapies in IHD and highlighted the transformative potential of advanced biomaterial strategies in cardiovascular medicine.

## Data Availability

The original contributions presented in the study are included in the article/Supplementary Material, further inquiries can be directed to the corresponding author.
