# Supplementary material for: Engineered extracellular vesicles for ischemic heart diseases: modification methods, targeted delivery strategies, and multi-modal therapies - A systematic review
Source: Front Cardiovasc Med. 2026 Jun 18;13:1868328. doi: 10.3389/fcvm.2026.1868328 (PMC13323252; doi:10.3389/fcvm.2026.1868328)
Supplement: Supplementary file 1 [file Datasheet1.docx]

**Supplementary Data**

Search Strategy：("engineered membrane vesicle*"[Title/Abstract] OR "engineered extracellular vesicle*"[Title/Abstract] OR "engineered vesicle*"[Title/Abstract] OR "engineered exosome*"[Title/Abstract] OR "nanovesicle*"[Title/Abstract] OR "nanoscale membrane vesicle*"[Title/Abstract] OR "nanoscale extracellular vesicle*"[Title/Abstract] OR "nanoscale vesicle*"[Title/Abstract] OR "nanoscale exosome*"[Title/Abstract]) AND ("myocardial ischemia"[MeSH Terms] OR "angina pectoris"[MeSH Terms] OR "myocardial infarction"[MeSH Terms] OR "myocardial ischemia"[Title/Abstract] OR "angina pectoris"[Title/Abstract] OR "myocardial infarction"[Title/Abstract] OR "coronary atherosclerotic heart disease"[Title/Abstract] OR "coronary disease*"[Title/Abstract] OR "coronary heart disease"[Title/Abstract] OR "CHD"[Title/Abstract] OR "ischemic heart disease*"[Title/Abstract] OR "angina"[Title/Abstract] OR "MI"[Title/Abstract])
